# Low Frequency Magnetic Fields Induce Autophagy-associated Cell Death in Lung Cancer through miR-486-mediated Inhibition of Akt/mTOR Signaling Pathway

**DOI:** 10.1038/s41598-017-10407-w

**Published:** 2017-09-18

**Authors:** Yujun Xu, Yizhong Wang, Anran Yao, Zhen Xu, Huan Dou, Sunan Shen, Yayi Hou, Tingting Wang

**Affiliations:** 1 The State Key Laboratory of Pharmaceutical Biotechnology, Division of Immunology, Medical School, Nanjing University, Nanjing, 210093 China; 2 Jiangsu Key Laboratory of Molecular Medicine, Nanjing, 210093 China

## Abstract

Low frequency magnetic fields (LF-MFs) can affect cell proliferation in a cell-type and intensity-dependent way. Previous study has reported the anti-tumor effect of LF-MFs in lung cancers. Our previous study also optimized the intensity and duration of LF-MFs to effectively inhibit the proliferation of lung cancer cells. However, the anti-tumor mechanism of LF-MFs remains unclear, which limit the clinical application of LF-MFs in anti-tumor therapy. Here, in a well-established Lewis Lung Cancer (LLC) mouse model, we found that LF-MFs inhibit tumor growth and induce an autophagic cell death in lung cancer. We also found that LF-MFs could up-regulate the expression level of miR-486, which was involved in LF-MFs activated cell autophagy. Furthermore, we found B-cell adaptor for phosphatidylinositol 3-kinase (BCAP) is a direct target of miR-486. miR-486 inhibit AKT/mTOR signaling through inhibiting expression of BCAP. Moreover, a decreased expression of miR-486 and an increased expression of BCAP were found in tumor tissues of lung cancer patients. Taken together, this study proved that LF-MFs can inhibit lung cancers through miR-486 induced autophagic cell death, which suggest a clinical application of LF-MFs in cancer treatment.

## Introduction

Lung cancer is the leading cause of cancer deaths worldwide, and approximately 80% of patients are non-small-cell lung cancer (NSCLC) among lung cancers^1^. The major clinical treatment in NSCLC is surgery, radiotherapy and chemotherapy^2,3^. However, patients with lung cancer still had a poor prognosis following these treatments. Therefore, alternative treatment, which could alter the growth of lung cancer cells, is very advantageous.

Many studies have investigated the anti-tumor effects of magnetic fields, with results that depend on multiple factors including filed frequency, intensity, exposure time and cell types^4,5^. Extremely Low Frequency Magnetic Fields (LF-MFs), which refer to magnetic fields with 3 Hz–30 Hz, have been shown to inhibit cancer cell proliferation in several studies^6,7^. LF-MFs can induce biological changes including improving immune function and regulate oncogenic or tumor suppressive gene expressions^8–10^. It’s proved that LF-MFs inhibit prostate cancer cell growth and induced cell cycle arrest by ROS production *in vitro* ^11^. Several *in vivo* studies proved the anti-tumor effects of LF-MFs with decreased tumor burden and longer survival time^9,10,12,13^. Our previous studies showed that LF-MFs (0.4 T, 7.5 Hz) can inhibit hepatocellular tumor and metastatic lung cancer *in vivo* ^14,15^. Of note, compared with chemotherapy and radiotherapy, safety is an advantage of LF-MFs. In a toxicity human study, patients with advanced cancer treated with LF-MFs showed no toxicity and adverse side effects. However, the detailed mechanisms behind the anti-tumor effects of LF-MFs remain unknown, which limits the clinical application of LF-MFs treatment.

Autophagy is a homeostatic degradative process which removes damaged organelles and maintains cell stability^16,17^. The role of autophagy in cancer development is controversial^18,19^. Autophagy was initially taken as a tumor-suppressive mechanism. Autophagy defects lead to chronic tissue damage and regeneration, which promote cancer development^20–22^. The critical autophagy gene ATG6/BECN1 was found lost in human prostate, ovarian and breast cancers^23–25^. In the process of autophagy, several autophagy-related genes (ATG) have been reported. Among them, ATG5 is conjugated with ATG12 to form ATG5-ATG12 complex and contributes to closure and elongation of autophagosomes in the generation of the microtubule-associated protein light chain 3 (LC3) family proteins^26–29^. Some other studies proved that autophagy promotes cancer through limiting stress responses and increasing cancer cell metabolism and survival^30–34^. Study by Marchesi *et al*., found specific intensity and frequency of LF-MFs can regulate the autophagy of neuroblastoma and alleviate the progression of neurodegenerative disease^35^. This data suggests a regulatory effect of LF-MFs on cell autophagy. However, how LF-MFs activate cell autophagy and whether this activation affect tumor development remains to be researched.

MicroRNAs (miRNAs) are small noncoding RNA molecules that regulate gene expression by translational repression or degradation of target mRNAs. miRNAs have emerged as major regulators of the initiation and progression of human cancers, including lung cancer. Furthermore, miRNAs play critical role in transcriptional control of autophagy^36^. Kong *et al*. found miR-423-3p-Bim axis promote cancer progression and activate oncogenic autophagy in gastric cancer^37^. miR-185 induce potent autophagy via AKT signaling in hepatocellular cancer^38^. Interestingly, previous study found LF-MFs treatments can modulate the expression of miR-30a *in vitro*, which in turn affects autophagy via Beclin1 expression. These studies suggest us miRNAs may attend in the anti-tumor effect of LF-MFs^35^.

In the present work, we explored the underlying anti-tumor mechanisms of LF-MFs using a well-established Lewis Lung Cancer (LLC) mouse model. We find LF-MFs induce an autophagic cell death in lung cancer cells. LF-MFs-induced cell death was associated with the down-regulation of miR-486. Moreover, miR-486 inhibit Akt/mTOR signaling through targeting the expression of BCAP (B-cell adaptor for phosphatidylinositol 3-kinase).

## Results

### LF-MFs suppress lung cancer growth *in vivo*

**Figure 1:**
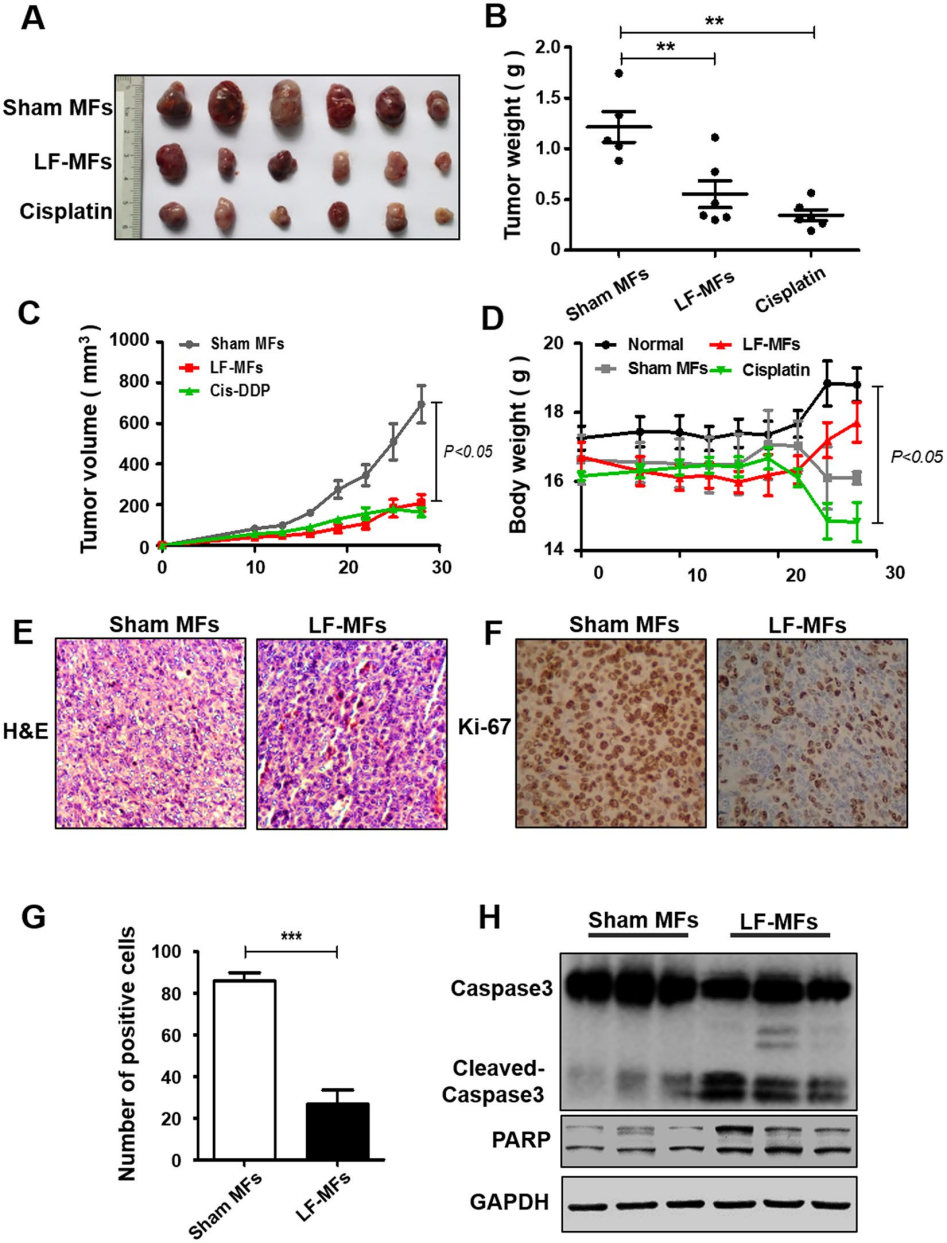
LF-MFs inhibit tumor growth in LLC mice model. Mice (n = 15, each group) were injected subcutaneously with Lewis lung cancer cells (1 × 10^6^ cells) and were treated with LF-MFs (0.4T, 7.5 Hz, 4 h/day) or Sham MFs for 35 days. (**A**) Representative tumors excised from mice treated with LF-MFs or Sham MFs. Cisplatin was used as a positive control. (**B**) LF-MFs treatment reduced the weight of LLC tumors. (**C**) LF-MFs treatment significantly inhibited the growth of LLC tumors. (**D**) LF-MFs treatment didn’t affect the body weight of mice. (**E**) H&E staining of tumor tissues in sham MFs or LF-MF treated mice. (**F**) Immunohistochemical staining for proliferation marker (Ki-67) in tumor tissues. (**G**) Ki-67 positive cells were quantified in tumors tissues. (**H**) Protein expression of Caspase3, cleaved-caspase3, and PARP were detected in tumor tissues from sham MFs group (n = 3) and LF-MFs group (n = 3) by Western blotting. Data from one representative experiment performed in triplicate. **P < 0.01 and ***P < 0.001.

We previously reported that LF-MFs prevent lung metastasis of melanoma. Here, the anti-tumor effect of LF-MFs was further confirmed on a Lewis lung carcinoma (LLC) subcutaneous injection mouse model. Cisplatin was used as a positive control. As shown in Fig. 1A–C, mice treated with LF-MFs (0.4 T, 7.5 Hz, 4 h/day) for 35 days had reduced tumor weight and tumor volume, which is comparable with mice treated with cisplatin. More importantly, no body weight loss was observed in LF-MFs-treated mice (Fig. 1D). H&E staining of the tumor tissues showed more cell mitosis in the group treated with Sham MF compared with the groups treated with LF-MFs (Fig. 1E). We then detected the numbers of Ki-67 positive cells, a tumor proliferation marker, by using immunohistochemistry. Compared with Sham MF treated tumors, expression of Ki-67 was significantly lower in LF-MFs treated tumors (Fig. 1F,G). To determine whether cell apoptosis was responsible for the reduction of proliferation, we examined two important apoptotic markers, the cleavage of casepase3 and poly ADP-ribose polymerase (PARP). LF-MFs treatment increased the protein levels of cleaved of casepase3 and PARP (Figs 1H and S1). These results validated that LF-MFs can suppress lung cancer growth *in vivo*.

### LF- MFs induced an autophagic cell death *in vivo* and *in vitro*

**Figure 2:**
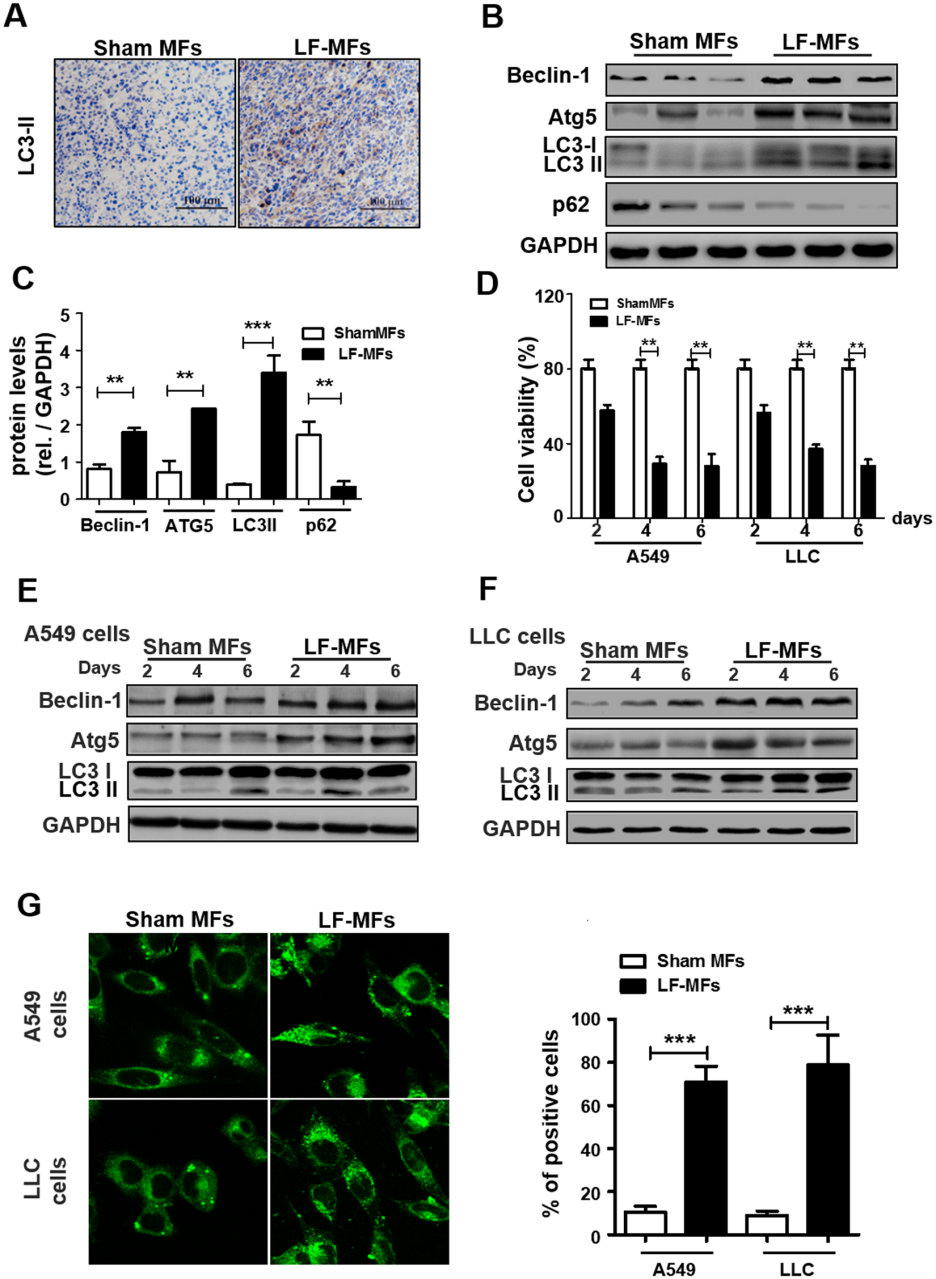
LF-MFs induce autophagy in tumor tissues and lung cancer cells. (**A**) Mice were treated as described in Fig. 1 and tumor tissues were acquired from each mouse. Expression of LC3II was detected using immunohistochemistry staining. (**B**,**C**) Protein expression of Beclin-1, ATG5, LC3B and p62 were detected in tumor tissues from sham MFs group (n = 3) and LF-MFs group (n = 3) by Western blotting. (**D**) A549 and LLC cells were treated with sham MFs or LF-MFs for different time intervals (2, 4, 6, days, 4 h/day). Cell activity was analyzed by using a cell counting kit-8 assay. (**E**,**F**) A549 and LLC cells were treated with sham MFs or LF-MFs for different time intervals (2, 4, 6, days, 4 h/day). (**G**) Fluorescent images of A549 and LLC cells transfected with the GFP-LC3 plasmids and treated with sham MFs or LF-MFs for 6d (original magnification × 1000). The images showed GFP-LC3 dots formation in LF-MFs-treated cells. All error bars indicate mean ± SEM. Experiments were repeated three times independently. ***P < 0.001.

To determine whether autophagy played a role in the anti-tumor process, expressions of specific marker of autophagy (LC3-II) were detected in tumor tissues. As showed in Fig. 2A, levels of LC3-II were increased in LF-MFs treated tumor tissues, compared with sham-MFs treated tumor tissues. More molecular markers in autophagy signaling were detected using western blotting. LF-MFs caused a remarkable increase in LC3-II, Atg5 and Beclin1 levels and blocked p62 degradation (Fig. 2B,C). Our results indicate that the autophagic flux in tumor tissues was activated upon LF-MFs treatment.

In consistent with our previous study, LF-MFs treatment can inhibit lung cancer cell proliferation *in vitro* (Fig. 2D). To assess whether autophagy contribute to this anti-tumor effect, human lung cancer A549 cells and mouse LLC cells were exposed to LF-MFs for different time intervals (2, 4, 6, days, 4 h/day). LF-MFs treatment up-regulate the expressions of Beclin1, Atg5 and LC3 II in both A549 cells and LLC cells (Fig. 2E,F and Fig. S2). We then performed a GFP-LC3 puncta-formation assay and a LC3 conversion assay, in which the punctate GFP-LC3 is indicative of autophagosomes. A549 and LLC cell lines were stably transfected with GFP-LC3. The transfection effect was determined by flow cytometry (Fig. S3). A549 and LLC cells that stably expressing GFP-LC3 fusion proteins were exposed to LF-MFs, the localization of GFP-LC3 was examined by confocal microscopy. As shown in Fig. 2G, LF-MFs significantly increased levels of LC-3II in both A549 and LLC cells. Together, these findings demonstrate that LF-MFs induced an autophagic cell death *in vivo* and *in vitro*.

### LF-MFs exposure modulates miR-486 expression in tumor cells

**Figure 3:**
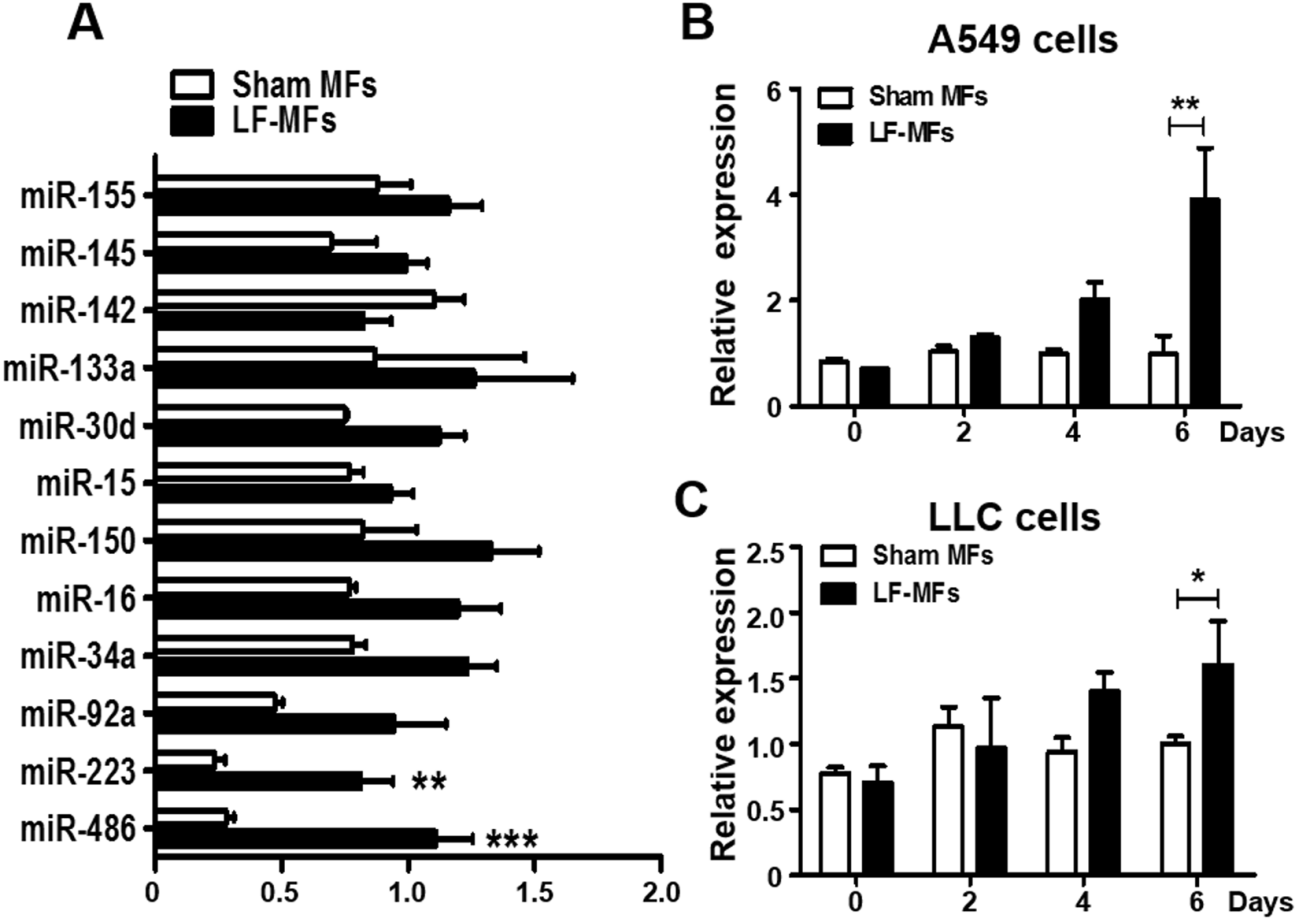
LF-MFs treatment up-regulate the expression of miR-486. (**A**) Mice were treated as described in Fig. 1 and tumor tissues were acquired from each mouse. miR-486 expression was significantly up-regulated by LF-MFs by using qRT-PCR. (**B**) A549 and LLC cells were treated with LF-MFs as described in Fig. 2C. qPCR analysis of the expression of miR-486 in A549 and LLC cells. The results are normalized to U6 snRNA levels. All error bars indicate mean ± SEM. Experiments were repeated three times independently. *P < 0.05, **P < 0.01 and ***P < 0.001.

Previous study showed that LF-MFs treatment can modulate the expression of miRNA. We therefore determined the effect of LF-MFs on several lung cancer related miRNAs in our mouse model. As shown in Fig. 3A, two miRNAs (miR-486, miR-223) were differentially expressed between LF-MFs and Sham MFs groups. Of note, miR-486 expression was significantly up-regulated in LF-MFs treated tumor tissues. Reduced miR-486 expression had been reported in lung cancer and miR-486 was considered as a tumor suppressor gene in NSCLC^39,40^. We also detected the expression of miR-486 in A549 and LLC cells with LF-MFs treatment. Compared to Sham MFs, miR-486 was significantly increased in both cells after exposure to LF-MFs for 6 days (Fig. 3B,C).

### Overexpression of miR-486 reduces cell proliferation and induces autophagy in lung cancer cells

**Figure 4:**
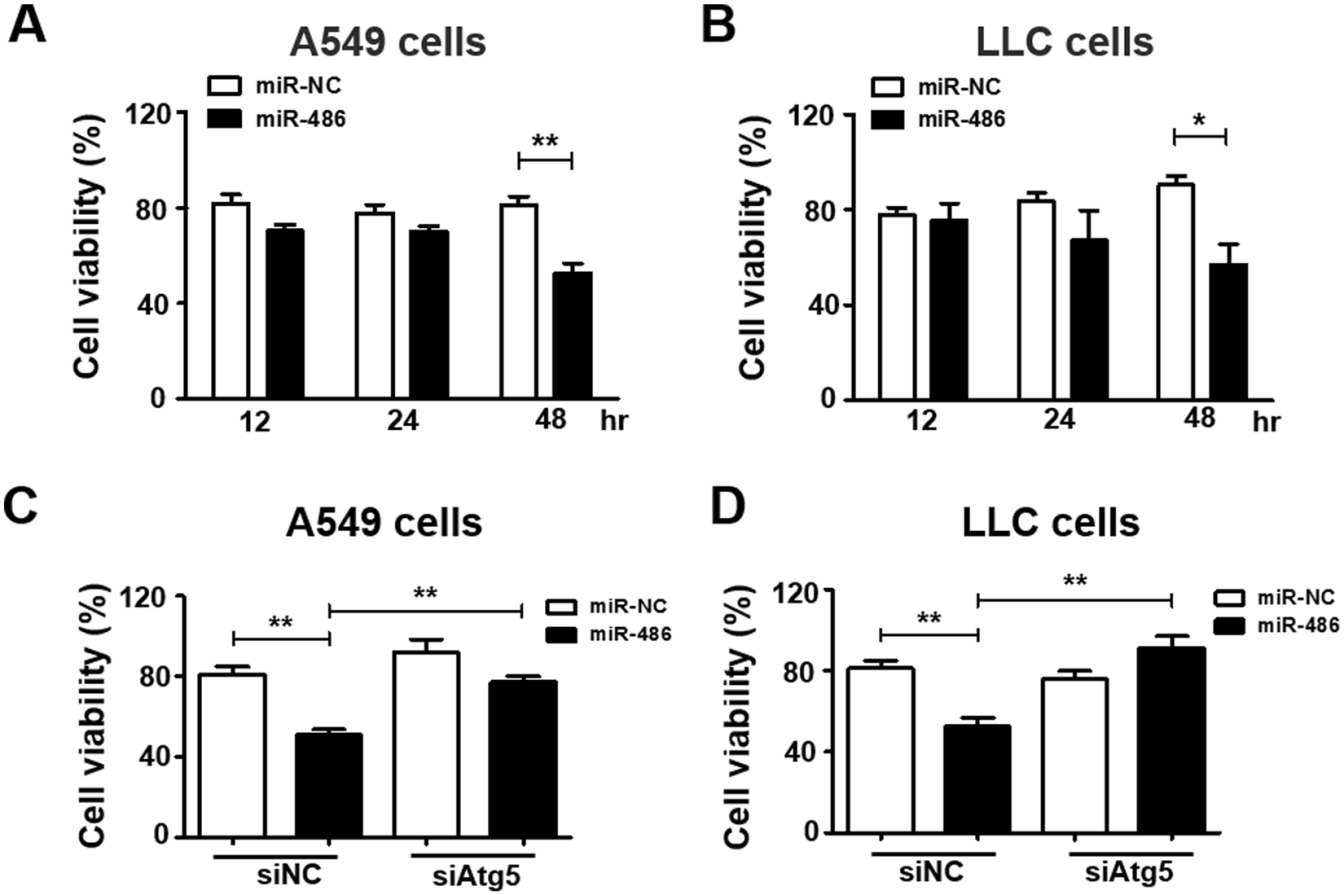
Up-regulation of miR-486 reduced the cell vitality via triggering autophagic cell death. (**A**,**B**) A549 and LLC cells were transfected with miR486 mimic and negative control. Cell activity was analyzed by using a cell counting kit-8 assay. (**C**,**D**) A549 and LLC cells were treated with non-targeting control small interfering RNA (siNC) or siRNAs specifc for autophagy-related Atg5. At 24 h after transfection, cells were transfected with miR-486 mimic and negative control for 48 h. Cell viability was measured in a cell counting kit-8 assay. All error bars indicate mean ± SEM. Experiments were repeated three times independently. *P < 0.05 and **P < 0.01.

To further investigate the functional consequences of increased miR-486 expression on tumor cells, we transfected lung cancer cells with miR-486 mimic or control (miR-NC). The transfection efficiency was measured and showed in Fig. S4A. In both A549 cells and LLC cells, cell proliferation rates of miR-486 transfected cells were reduced compared with cells transfected with negative control 48 h after transfection (Fig. 4A,B). To confirm the role of autophagy in LF-MFs induced cell death, siAtg5, one of the most critical proteins in autophagy process, which was able to inhibit autophagy, was transfected to lung cancer cells. The silencing efficiency in two cells was measured and showed in Fig. S4B–E. We found miR-486 induced cell death could be reversed by siAtg5 in both cells (Fig. 4C,D). These results imply that autophagy, at least partially, contributes to miR-486-induced cell death.

### LF-MFs activate cell autophagy via up-regulating miR-486 expression

**Figure 5:**
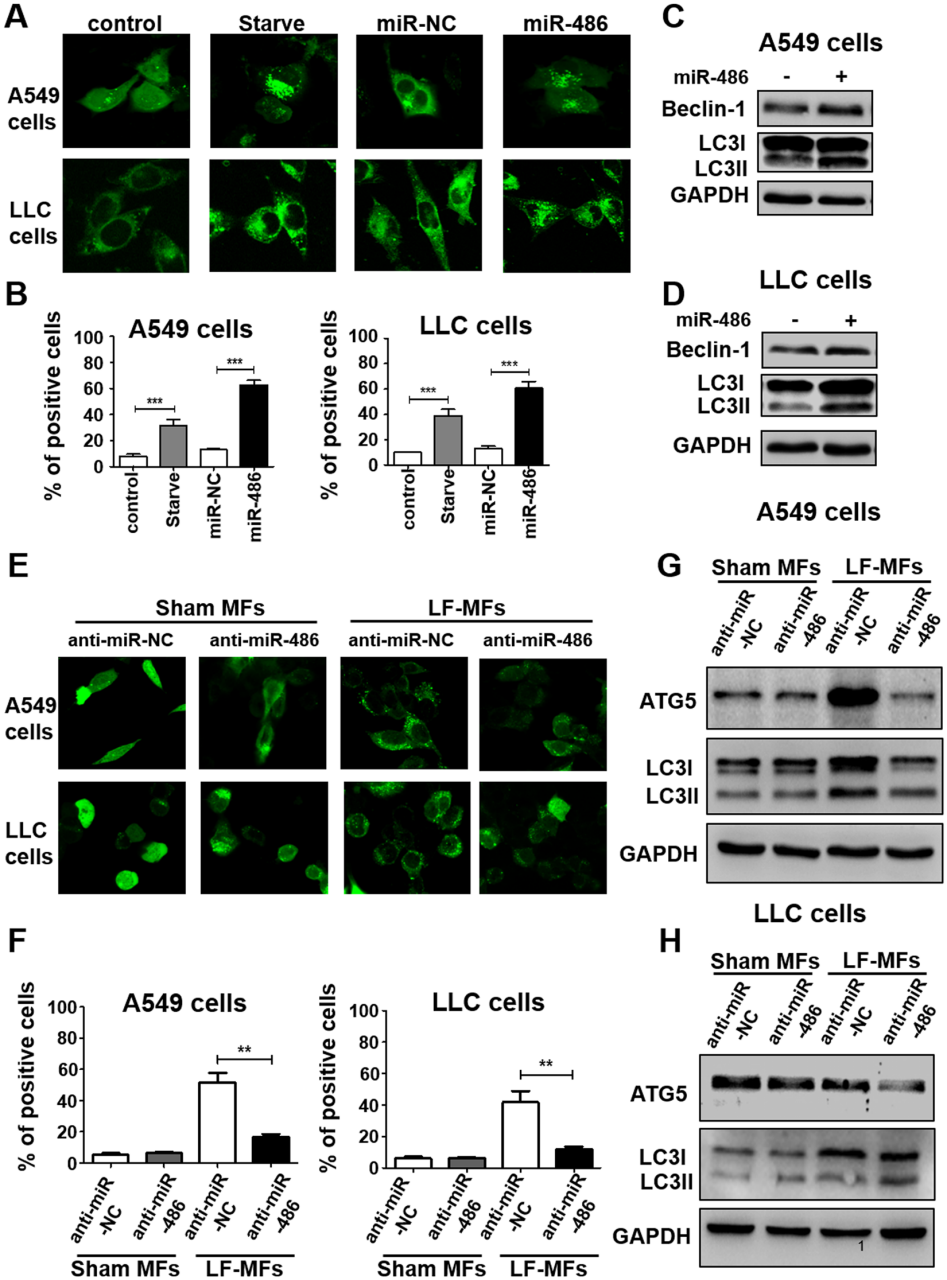
miR-486 is required for LF-MFs-induced cell autophagy. (**A**) A549 and LLC cells were transfected with miR-486 mimic or negative control together with GFP-LC3 plasmid. Cells transfected with GFP-LC3 plasmid only were taken as a blank control. Cells under starvation were taken as a positive control. (**B**) Number of GFP-LC3 puncta per transfected cell representing autophagosomes was quantified. (**C**,**D**) A549 and LLC cells were treated as described in (**A**) and were subjected to western blot analysis with the following antibodies: Beclin-1, LC3-II and GAPDH. (**E**) Cells were co-transfected with miR-486 inhibiter or negative control together with GFP-LC3 plasmid. After 48 hours, cells were treated with sham MFs or LF-MFs. (**F**) Number of autophagosomes was quantified. (**G**,**H**) A549 and LLC cells were treated as described in (**E**) and were subjected to western blot analysis with the following antibodies: Atg5, LC3-II, and GAPDH. All error bars indicate mean ± SEM. Experiments were repeated three times independently. **P < 0.01 and ***P < 0.001.

Given that LF-MFs act as an autophagy inducer in lung cancer and LF-MFs can regulate miRNA expression, we then confirm the function of miRNA as a regulator of autophagy. miR-486 was transfected into A549 and LLC cells that stably expressing GFP-LC3 fusion protein. Starvation was used as a positive control. As shown in Fig. 5A,B, overexpression of miR-486 significantly increased punctate GFP-LC3. miR-486 overexpression can also increase the protein level of LC3 II and Beclin-1 in both cells (Figs 5C,D and S5A,B).

We have already proved that LF-MFs have an important influence in autophagy and miR-486 expression. In addition, miR-486 could promote autophagy. We then wonder to know whether LF-MFs induced autophagy via miR-486. As shown in Fig. 5E,F, knockdown of miR-486 with siRNA suppressed the LF-MFs-induced punctate GFP-LC3 formation. Anti-miR-486 also reverse LF-MFs induced LC3-II and ATG5 levels (Figs 5G,H, and S5C,D). Together, the findings suggest that miR-486 is required for LF-MFs triggered autophagy.

### B-cell adaptor for phosphatidylinositol 3-kinase (BCAP) is a direct target of miR-486

**Figure 6:**
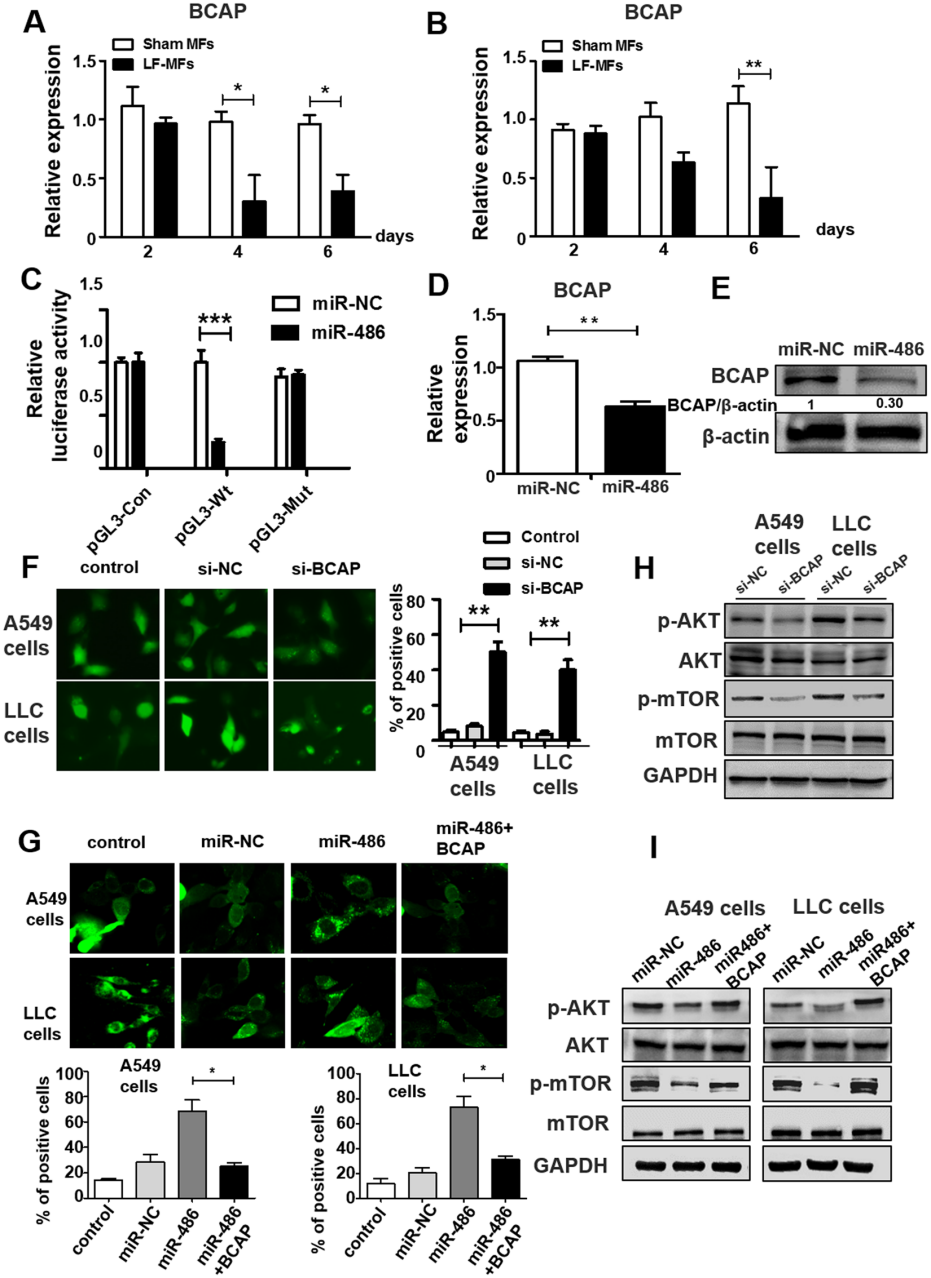
miR-486 directly regulate post-transcriptional expression of BCAP. (**A**,**B**) A549 and LLC cells were treated as described in Fig. 2C. Expression levels of BCAP were detected by qRT-PCR. (**C**) Luciferase reporters containing wild-type (WT) or mutant (MUT) miR-486 binding sites in the BCAP 3′-UTR were co-transfected into 293 T cells along with control luciferase reporter plasmid, miR-NC, miR-486 mimic for 48 hours. (**D**–**E**) qRT-PCR analysis of BCAP mRNA levels and Western blot analysis of BCAP protein levels in A549 cell treated with miR-486 mimic. (**F**) A549 and LLC cells were co-transfected with GFP-LC3 plasmid, siNC or siBCAP for 48 h. Number of autophagosomes was quantified. (**G**) A549 and LLC cells were transfected with GFP-LC3 plasmid, miR-NC, miR-486 mimic or co-transfected with miR-486 mimic and BCAP plasmid. Number of autophagosomes was quantified. (**H**) A549 and LLC cells transfected with siNC or siBCAP for 48 h. Western blot analysis of phospho-AKT, AKT, phospho-mTOR, mTOR and GAPDH expression. (**I**) A549 or LLC cells transfected with miR-NC, miR-486 mimic or co-transfected with miR-486 and BCAP plasmid. Western blot analysis of phospho-AKT, AKT, phospho-mTOR, mTOR and GAPDH expression. All error bars indicate mean ± SEM. Experiments were repeated three times independently. **P < 0.01.

We searched target prediction database for putative target genes of miR-486. Target prediction algorithms using target scan (www.targetscan.org) or DianaLab (http://diana.cslab.ece.ntua.gr) revealed that BCAP is a potential target of miR-486 (Fig. S6). It was also reported that AKT activation by B cell receptor cross-linking was dependent on BCAP^41,42^. However, whether BCAP is an important player in cancers is unknown. We first detected the expression of BCAP in two lung cancer cells. We found that BCAP mRNA levels decreased significantly in LF-MFs treated cells compare with Sham MF treated cells (Fig. 6A,B). To verify whether BCAP was the direct target of miR-486, the 3′UTR of BCAP was sub-cloned downstream of the flue open reading frame in PGL3 vector. The 293T cells were co-transfection of miR-486 mimic with the luciferase reporter gene linked to the wild-type segment of the BCAP 3′UTR strongly repressed luciferase activity, while the luciferase activity of the mutant was significantly rescued (Fig. 6C). Moreover, the mRNA and protein expression level of BCAP were also significantly inhibited in miR-486-transfected A549 cell (Fig. 6D,E). These results demonstrated that miR-486 specifically regulates BCAP expression at the post-transcriptional level. The role of BCAP in autophagy was further determined using siRNA targeting BCAP (Fig. S6B,C). We found BCAP siRNA could induce autophagic activity compared with control siRNA (Fig. 6F). To evaluate if BCAP re-addition could revert the autophagic activity in miR-486 cells, a BCAP plasmid was designed and was co-transfected with miR-486 mimic into A549 cells and LLC cells that stably expressing GFP-LC3 fusion protein. We found miR-486 induced autophagic activity could be suppressed by re-adding BCAP in both cells (Fig. 6G).

### miR-486 inhibit AKT/mTOR pathway via BCAP

It has been reported that AKT/mTOR is a key signaling pathway, which is involved in the early triggering of autophagy. Suppression of AKT decreases mTOR activity and promotes autophagy^43^. In our study, the expression levels of p-AKT and p-mTOR were down-regulated in lung cancer cells transfected with si-BCAP or miR-486 mimic (Fig. 6H,I). We further found miR-486 inhibited p-AKT and p- mTOR levels could be rescued by re-adding BCAP in both cells (Fig. 6I). Together, this data suggests that miR-486 inhibit AKT/mTOR pathway via targeting BCAP in lung tumor cells.

### Correlation of miR-486 and autophagy in tumor tissues of NSCLC patients

**Figure 7:**
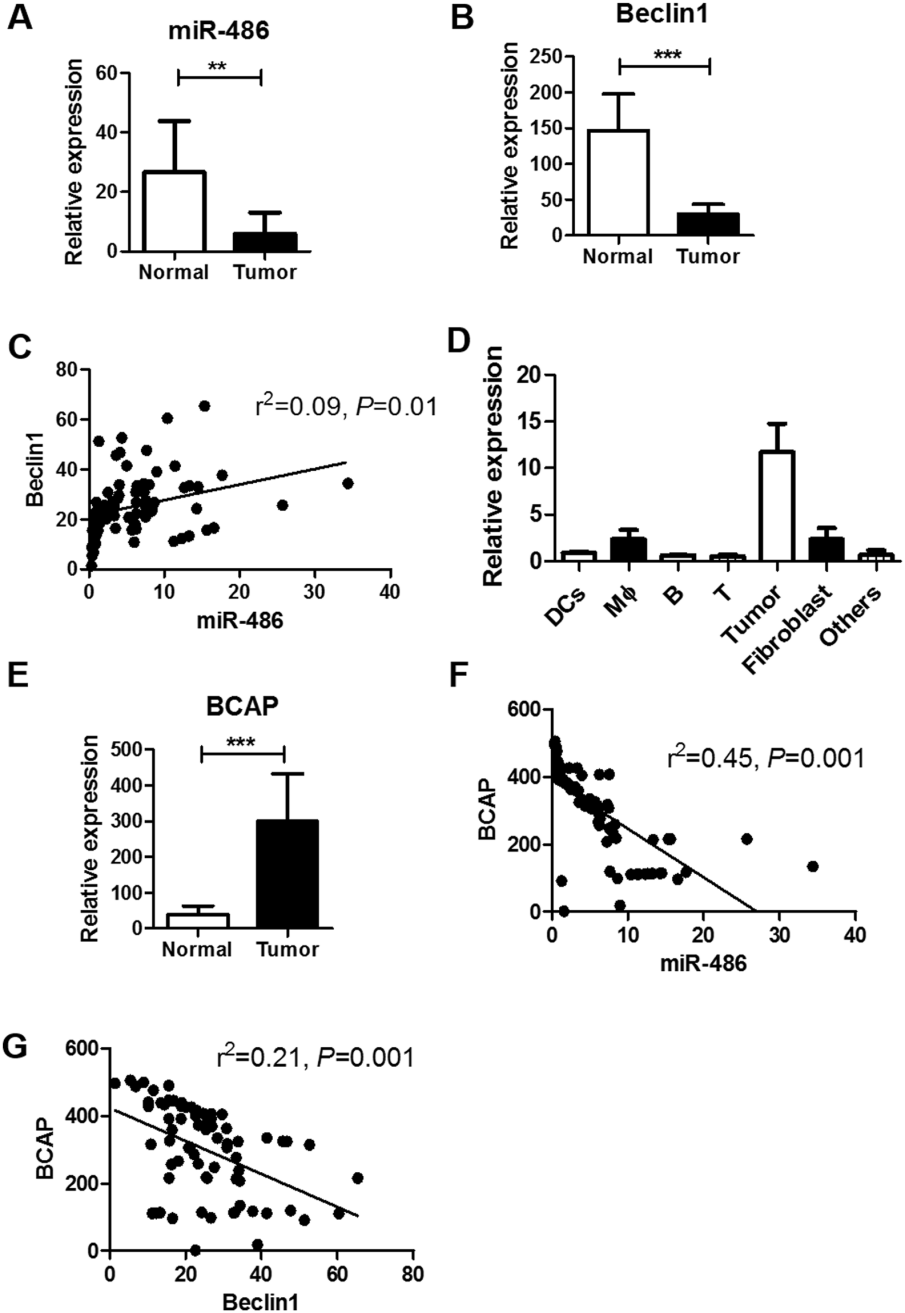
Correlation of miR-486 and autophagy in tumor tissues of NSCLC patients. (**A**,**B**) 71 patients diagnosed with NSCLC were recruited. Tumor tissues and paratumoral normal tissues were acquired from each patient. Expression of miR-486 and Beclin1 in tumor tissues were detected using qPCR. (**C**) Correlation between expression of miR-486 and Beclin1. (**D**) Different cell types were isolated from lung tumor tissues using magnetic cell sorting. Expressions of miR-486 in these cells were determined by qPCR. (**E**) Expression of BACP was detected in tumor tissues and normal tissues using qPCR. (**F**) Correlation between expression of miR-486 and BACP. (**G**) Correlation between expression of Beclin1 and BACP. All error bars indicate mean ± SEM. Experiments were repeated three times independently. **P < 0.01 and ***P < 0.001.

Autophagy has been related with malignant behaviors and clinical outcome in NSCLC^44^. We further confirmed the relationship between miR-486 and autophagy in tumor tissues of NSCLC patients. 71 patients diagnosed with NSCLC were recruited and the clinical characteristics were listed in Table 1. Tumor cells and paratumoral normal tissues were acquired from each patient. Compared with normal tissues, there were decreased expressions of miR-486 and Beclin1 in tumor tissues (Fig. 7A,B). Regression analysis showed a positive correlation between expression of miR-486 and Beclin1 (Fig. 7C). To distinguish the origin of miR-486 in lung tumor tissues, different types of cells were isolated from tumor tissues by magnetic cells sorting and the expression of miR-486 was determined. We found miR-486 was mostly expressed in tumor cells (Fig. 7D). We also detected the mRNA expression of BACP in lung cancer tissues. As shown in Fig. 7E, higher expression of BACP was found in tumor tissues, compared with normal tissues. Regression analysis also showed a negative correlation between expression of BACP and miR-486 (Fig. 7F), and a negative correlation between expression of BACP and Beclin1 (Fig. 7G).

**Table 1:** Clinicopathological characteristics of NSCLC patients.

Characteristics	Patient number (%)
Gender	
Female	32 (48.6%)
Male	39 (51.4%)
Age (years)	
<60	40 (43.1%)
≥60	31 (56.9%)
Smoking Status	
Nonsmoker	19(22.2%)
Ever-smoker	49 (76.3%)
Unknown	3 (1.5%)
Histology	
Adenocarcinoma	71 (100%)
Others	0 (0%)
Stage	
I	0 (0%)
II	31 (36.1%)
III	40 (63.9%)
IV	0 (0%)

## Discussion

In this study, we find LF-MFs inhibit tumor growth and induce an autophagic cell death in lung cancer. We also find that LF-MFs could up-regulate the expression level of miR-486, which is involved in LF-MFs activated cell autophagy. Furthermore, BCAP is proved to be a direct target of miR-486. miR-486 inhibit AKT/mTOR signaling through inhibiting expression of BCAP. Moreover, a decreased expression of miR-486 and an increased expression of BCAP were found in tumor tissues of lung cancer patients. Taken together, this study proved that LF-MFs can inhibit lung cancers through miR-486 induced autophagic cell death, which suggest a clinical application of LF-MFs in cancer treatment.

The biological effects of MFs in the development of cancer have been discovered step by step. Firstly, parental occupational exposure to LF-MFs adds no risk of childhood acute lymphoblastic leukaemia^45^. Then appropriately application of LF-MFs was found to inhibit gastric cancer, liver cancer and lung cancer metastatic *in vitro* and *in vivo* ^9,14,15,46^. Furthermore, LF-MFs showed no toxicity and adverse side effects in patients with advanced cancer^47^. The mechanism of anti-tumor effect of LF-MFs includes inducing tumor cell apoptosis, promoting ROS production and regulating anti-tumor immune system^48^. Here, we optimize the intensity and duration of LF-MFs, and found LF-MFs (0.4T, 7.5 Hz, 4 h/day) can significantly inhibit lung cancer growth in LLC mouse model, which is comparable to cisplatin. Of note, LF-MFs did not induce weight loss as cisplatin during treatment. Therefore, LF-MFs showed a potential prospect in clinical application.

Autophagy has a two-way role in the process of tumor formation, which may depend on cell type or mouse model^49–51^. On one hand, autophagy may prevent excessive protein degradation and can be inhibited by different oncoproteins, such as AKT, PI3K and Bcl-1. On the other hand, persistent activation of autophagy cause autophagic programmed cell death or apoptosis^19^. Autophagic cell death (ACD) is characterized by the accumulated cytoplasmic vacuolization, the absence of chromatin condensation, LC3 lipidation and caspase-independent apoptosis^52^. Furthermore, there is a crosstalk between autophagy and apoptosis. Autophagy can play a positive and negative role in promoting apoptosis^53,54^. In our study, we provide evidence that LF-MFs can induce ACD and inhibit lung cancer for the first time. Autophagic cell death was found in both LLC tumor tissues *in vivo* and lung cancer cells *in vitro*.

miRNAs have emerged as major regulators of the initiation and progression of human cancers, including lung cancer. Recently, several miRNAs were found to regulate autophagy pathways in NSCLC. For example, miR-17 downregulation contributes to paclitaxel resistance of lung cancer cells through altering beclin1 expression^55^. MiR-143 inhibits cell proliferation by targeting autophagy-related 2B in NSCLC^56^. MiR-638 promotes melanoma metastasis and protects melanoma cells from apoptosis and autophagy^57^. Here, we found LF-MFs treatment can up-regulate expression of miR-223 and miR-486. However, we did not perform experiment on miR-223 since the role of miR-223 on lung cancer is controversial. It was reported that miR-223 is a tumor suppressor miRNA, which could suppress LLC by targeting insulin-like growth factor-1 receptor. Lower expression level of miR-223 was observed in LLC tissue than normal tissues^58^. However, miR-223 was significantly up-regulated in human lung cancer A549 cells compared with BEAS-2B cells^59^. NSCLC patients contain higher level of miR-223 than that from healthy subjects^60^. We also found different basal levels of miR-223 in our preliminary experiment. Therefore, we focus on miR-486 in our study. We proved that miR-486 can affect cell autophagy through targeting BCAP and AKT pathway. miR-486 is a tumor suppressive gene, which was associated with insulin growth factor signaling and had an effect in tumor progression and metastasis^39,40,61^. In consistent with previous study, we found decreased expression of miR-486 in tumor tissues, compared with normal tissues. We also proved significant correlation between miR-486 and BCAP, and correlation between miR-486 and Beclin1 in tumor tissues. These data suggest miR-486 may regulate autophagic cell death through BCAP in lung cancer patients, which can be a potential target for LF-MFs treatment.

## Materials and Methods

### Animals model

Animal studies were approved by Medical School for Animal Use and Care Committee of Nanjing University in accordance with the guideline of the US NIH. 4–6 week-old female C57BL/6 mice were purchased from the Model Animal Research Center of Nanjing University. Mice were cultured under specific pathogen-free conditions with the temperature of 24 °C, standard rodent chow and water. For the establishment of xenografts, Lewis lung cancer cells (1 × 10^6^ cells/40 µl) were injected subcutaneously into the left armpits of mice. Mice were randomly divided into three group when tumors reached a volume of 50 to 100 mm^3^: Sham LF-MFs group (tumor mice were not exposed to LF-MFs, n = 15), LF-MFs group (tumor mice were exposed to LF-MFs, n = 15) and cisplatin group (tumor mice were administrated with 5 mg/kg of Cisplatin once every three days, n = 15). On day 35, mice were sacrificed. Tumor volume was measured every 3 days by a vernier caliper and calculated using the formula: volume = length × width^2^ × π/6. Tumors with metastasis were measured and prepared for further analysis.

### Cell cultures

293T cells and Lung cancer cell lines (A549, LLC) were obtained from the Shanghai Institute of Cell Biology (Shanghai, China). Cells were cultured in DMEM medium (Gibco, Carlsbad, CA) with 10% fetal bovine serum (Gibco, Carlsbad, CA). All cell lines were incubated at 37 °C in a water-saturated condition with 5% CO_2_.

### miRNA Transfection

A549 and LLC cells were transfected with Synthetic pre-miR-48 or scrambled negative control RNAs were purchased from Ribobio (Guangzhou, China) using Lipofectamine 2000 (Invitrogen, USA), according to the manufacturer’s instructions. For miRNA silencing, an inhibitor of miR-486 or negative inhibitor control (Ribobio) were transfected into the two cells using Lipofectamine 2000 (Invitrogen) according to the manufacturer’s instructions.

### Small Interfering RNAs (siRNAs) and Overexpression of BCAP

siRNAs targeting Atg5 and BCAP were purchased from Ribobio (Guangzhou, China). Cells were transfected with 50 nM of siRNA or non-targeting siRNA controls using Lipofectamine 2000 reagent (Invitrogen) according to the manufacturer’s instructions and re-transfected after 24 hours. For overexpression of BCAP in two cells, cells were cotransfected with miR-486 or expression plasmid for BCAP from Genechem (Shanghai, China) using Lipofectamine 2000 reagent (Invitrogen). Cells were harvested 48 h after transfection for subsequent tests. The siRNA sequences were as follows:

Non-targeting control siRNA; 5′-ACGUGACACGUUCGGAGAAUU-3′;

Atg5: 5′-AUCCCAUCCAGAGUUGCUUGUGAUC-3′;

BCAP: 5′-TGTCATCCAGCTTACATCTCACAC-3′. The BCAP mRNA and ATG5 protein expression levels were assessed by qRT-PCR and Western blotting.

### Real-time quantitative PCR analysis

Total RNAs were extracted from tissues and cultured cells with TRIzol Reagent (Invitrogen, USA) according to the manufacturer’s instructions. A total of 1 μg RNA was used as the template for single strand cDNA synthesis. Q-PCR for GAPDH, miRNAs and SiRNAs was performed on an ABI Step One Plus Detection System (Applied Biosystems, USA) using SYBR green dye (Bio-Rad, USA). The reaction conditions were 95 °C for 10 min, then 40 cycles of 95 °C for 15s, 60 °C for 30s, and 72 °C for 30s. All reactions were run in triplicate. Gene expression levels were normalized to GAPDH. The sequences of the primers for gene expression are listed in Table S1.

### GFP-LC3 stable cell lines and quantitative GFP-LC3 analyses

A549 and LLC GFP-LC3 stable cell line was established by transient transfection of GFP-LC3 plasmid using Lipofectamine 2000 reagent (Invitrogen). A549 GFP-LC3 or LLC GFP-LC3 stable cell lines were transfected with individual miRNAs, siRNAs or treated with LF-MFs. GFP-LC3 punctum formation in was determined by capturing images using Olympus FV1000 confocal microscope (Olympus, Tokyo, Japan).

### Construction of luciferase plasmids and reporter assay

The 3′UTRs of BCAP containing the predicted miR-486 binding sequence was PCR amplified and inserted into the XbaI site in the luciferase reporter pGL3-Report vector (Promega, Biotech Co., Ltd.). Mutant reporter constructs where the miR-486 binding sites were mutated were generated by PCR-based mutagenesis. Cells were co-transfected with 200 ng of pGL-Report vectors containing either the wild-type or mutated BCAP 3′UTR, and miR-486-specific precursor (pre-miR-486) or pre-miR-control (premiR-NC) using Lipofectamine 2000 reagent (Invitrogen). The pGL3-control vector was also included as an internal control for transfection efficiency. After 48 hours, the luciferase activity was determined using the dual luciferase reporter assay system (Promega, Biotech Co., Ltd.). The relative reporter activity was obtained by normalization to the Renilla luciferase activity. All experiments were performed in triplicate.

### Western blotting and Antibodies

Tumor tissue or cell lines were lysed in a buffer containing 50 mM Tris-Cl pH 8.0, 150 mM NaCl, 0.02%NaN3, 0.1%SDS, 100 mg/ml phenyl-methylsufonyl fluoride (PMSF), 1 mg/ml Aprotinin, 1%Triton. 10 μg/mL aprotinin, 10 μg/mL leupeptin, 1 mM dithiothreitol, 1 mM paranitrophenyl phosphate, and 0.1 mM Na_3_VO_4_ were added as protease and phosphatase inhibitor. After centrifugation, protein samples were subjected to 10% SDS-PAGE and transferred onto PVDF membranes (Roche, Mannheim, Germany). The membranes were blocked in TBST (1 mM Tris-HCl, pH 7.4, 150 mM NaCl, 0.05% Tween-20) containing 5% BSA for 1.5 h and subsequently incubated overnight at 4 °C with diluted primary antibodies against antibodies. All antibodies were used at a dilution of 1:1,000 unless specified otherwise. Mouse monoclonal antibodies were from: R&D (BCAP, MAB4857), Thermo Fisher Scientific (GAPDH, MA5-15738-BTIN), Rabbit polyclonal antibodies were from: CellSignalling (ATG5-2630, p-mTOR-2974), Bioword Technology (AKT-BS1810), Rabbit monoclonal antibodies were from: Millipore (anti-mTOR-04-385), Proteintech (p62-18420-1-AP, β-actin-20536-1-AP), CellSignalling (Beclin-1-3495, LC3B-3868, Caspase-3-9665, Cleaved Caspase-3-9664, PARP-9532, p-AKT-4060).

### Immunohistochemistry analysis

Tissue samples were fixed with 4% paraformaldehyde, dehydratedin ethanol, and embedded in paraffin. The slides were then deparaffinized. For immunohistochemical staining, the slides were treated with 3% H2O2, followed by blocking with 10% horse serum in PBS for an hour. The samples were stained with anti-Ki-67and anti-LC3B (1:100 dilution in 0.5% bovine serum albumin in PBS) for 2 h, followed by a biotinylated secondary antibody for another30 min, and then streptavidin-HRP for 20 min, with PBS washes after each incubation. The slides were developed by incubation with the 3,30-diaminobenzidine (DAB) substrate kit. The images from Ki-67and anti-LC3B staining were collected from five fields per section under 200x magnifications and processed using NIH ImageJ software. Staining intensity was measured from five randomly selected views using Image ProPlus software (Media Cybernetis, Rockville, MD). Six mice from each group were included for statistical analysis.

### Cell vitality assay

A549 and LLC cells placed in 96-well plates. Cells were treated with Sham MFs or LF-MFs or co-transfected with miRNAs and siRNAs and cultured at 37 °C, 5% CO_2_ condition. With different culture time, cell supernatants were changed to fresh medium with 10% CCK8 (Dojindo Laboratories, Japan). The absorption data at 450 nm were detected 30 minutes or longer later using microplatereader.

### Patient samples

71 NSCLC patients who received treatment in Nanjing Chest Hospital between Jan 2007 to Dec 2012 were recruited in this study. Tumor tissues were acquired when patients receive operation. This study was carried out in accordance with the Declaration of Helsinki and the privacy of patients was strictly protected. Experimental protocols were approved by the medical research ethical committee of Nanjing Chest Hospital. Written informed consent was obtained from all participants. To isolate primary cells from tumor tissues, fibroblast cells were firstly isolated by repeated adherence. Other cells were isolated by magnetic cells sorting kit (DCs: 130-090-506; Macrophagy: 130-049-601; T cells: #130-050-101; B cell: 130-091-151; Tumor cells: 130-092-234) following the manuscript introduction (Miltenyi Biotec, Germany).

### Statistical analysis

Results were presented as mean ± SEM. Student’s t-test was used to compare between two groups. One-way ANOVA analysis of variance was used to compare among three or more groups. Correlation significance was determined by using linear regression. A p value of <0.05 was considered significant, and survival curves were performed with GraphPad Prism soft-ware (version 5.01, San Diego, CA, USA).

## Electronic supplementary material

supplementaryinformation

## References

[1] Jemal A (2011). Global cancer statistics. CA: a cancer journal for clinicians.

[2] Patel AP (2014). National patterns of care and outcomes after combined modality therapy for stage IIIA non-small-cell lung cancer. Journal of thoracic oncology: official publication of the International Association for the Study of Lung Cancer.

[3] Heist RS, Engelman JA (2012). SnapShot: non-small cell lung cancer. Cancer cell.

[4] Edelman NB (2015). No evidence for intracellular magnetite in putative vertebrate magnetoreceptors identified by magnetic screening. Proceedings of the National Academy of Sciences of the United States of America.

[5] Eder SH (2012). Magnetic characterization of isolated candidate vertebrate magnetoreceptor cells. Proceedings of the National Academy of Sciences of the United States of America.

[6] Mattsson MO, Simko M (2012). Is there a relation between extremely low frequency magnetic field exposure, inflammation and neurodegenerative diseases? A review of *in vivo* and *in vitro* experimental evidence. Toxicology.

[7] Zimmerman JW (2012). Cancer cell proliferation is inhibited by specific modulation frequencies. British journal of cancer.

[8] Crocetti S (2013). Low intensity and frequency pulsed electromagnetic fields selectively impair breast cancer cell viability. PloS one.

[9] Nie Y (2013). Low frequency magnetic fields enhance antitumor immune response against mouse H22 hepatocellular carcinoma. PloS one.

[10] Tatarov I (2011). Effect of magnetic fields on tumor growth and viability. Comparative medicine.

[11] Koh EK (2008). A 60-Hz sinusoidal magnetic field induces apoptosis of prostate cancer cells through reactive oxygen species. International journal of radiation biology.

[12] Jimenez-Garcia MN (2010). Anti-proliferative effect of extremely low frequency electromagnetic field on preneoplastic lesions formation in the rat liver. BMC cancer.

[13] Bouchlaka MN (2012). Mechanical disruption of tumors by iron particles and magnetic field application results in increased anti-tumor immune responses. PloS one.

[14] Wang T (2011). Involvement of midkine expression in the inhibitory effects of low-frequency magnetic fields on cancer cells. Bioelectromagnetics.

[15] Nie Y (2013). Effect of low frequency magnetic fields on melanoma: tumor inhibition and immune modulation. BMC cancer.

[16] Wu X (2016). Autophagy regulates Notch degradation and modulates stem cell development and neurogenesis. Nature communications.

[17] Marino G (2011). Longevity-relevant regulation of autophagy at the level of the acetylproteome. Autophagy.

[18] Schmeisser H (2013). Type I interferons induce autophagy in certain human cancer cell lines. Autophagy.

[19] White E (2015). The role for autophagy in cancer. The Journal of clinical investigation.

[20] Mathew R (2009). Autophagy suppresses tumorigenesis through elimination of p62. Cell.

[21] Karantza-Wadsworth V (2007). Autophagy mitigates metabolic stress and genome damage in mammary tumorigenesis. Genes & development.

[22] Mathew R (2007). Autophagy suppresses tumor progression by limiting chromosomal instability. Genes & development.

[23] Liang XH (1999). Induction of autophagy and inhibition of tumorigenesis by beclin 1. Nature.

[24] Choi AM, Ryter SW, Levine B (2013). Autophagy in human health and disease. The New England journal of medicine.

[25] Aita VM (1999). Cloning and genomic organization of beclin 1, a candidate tumor suppressor gene on chromosome 17q21. Genomics.

[26] Fader CM, Colombo MI (2009). Autophagy and multivesicular bodies: two closely related partners. Cell death and differentiation.

[27] Shintani T, Klionsky DJ (2004). Autophagy in health and disease: a double-edged sword. Science.

[28] Dunn WA (1994). Autophagy and related mechanisms of lysosome-mediated protein degradation. Trends in cell biology.

[29] Martinez J (2011). Microtubule-associated protein 1 light chain 3 alpha (LC3)-associated phagocytosis is required for the efficient clearance of dead cells. Proceedings of the National Academy of Sciences of the United States of America.

[30] Lock R (2011). Autophagy facilitates glycolysis during Ras-mediated oncogenic transformation. Molecular biology of the cell.

[31] Guo JY (2011). Activated Ras requires autophagy to maintain oxidative metabolism and tumorigenesis. Genes & development.

[32] Yang S (2011). Pancreatic cancers require autophagy for tumor growth. Genes & development.

[33] Lock R, Kenific CM, Leidal AM, Salas E, Debnath J (2014). Autophagy-dependent production of secreted factors facilitates oncogenic RAS-driven invasion. Cancer discovery.

[34] Degenhardt K (2006). Autophagy promotes tumor cell survival and restricts necrosis, inflammation, and tumorigenesis. Cancer cell.

[35] Marchesi N (2014). Autophagy is modulated in human neuroblastoma cells through direct exposition to low frequency electromagnetic fields. Journal of cellular physiology.

[36] Fullgrabe J, Klionsky DJ, Joseph B (2014). The return of the nucleus: transcriptional and epigenetic control of autophagy. Nature reviews. Molecular cell biology.

[37] Kong P (2017). The microRNA-423-3p-Bim Axis Promotes Cancer Progression and Activates Oncogenic Autophagy in Gastric Cancer. Molecular therapy: the journal of the American Society of Gene Therapy.

[38] Zhou, L. *et al*. MicroRNA-185 induces potent autophagy via AKT signaling in hepatocellular carcinoma. *Tumour biology: the journal of the International Society for Oncodevelopmental Biology and Medicine* **39**, 1010428317694313, 10.1177/1010428317694313 (2017). 10.1177/1010428317694313 28240051

[39] Peng Y (2013). Insulin growth factor signaling is regulated by microRNA-486, an underexpressed microRNA in lung cancer. Proceedings of the National Academy of Sciences of the United States of America.

[40] Wang J (2014). Downregulation of miR-486-5p contributes to tumor progression and metastasis by targeting protumorigenic ARHGAP5 in lung cancer. Oncogene.

[41] Qin S, Chock PB (2003). Implication of phosphatidylinositol 3-kinase membrane recruitment in hydrogen peroxide-induced activation of PI3K and Akt. Biochemistry.

[42] Castello A (2013). Nck-mediated recruitment of BCAP to the BCR regulates the PI(3)K-Akt pathway in B cells. Nature immunology.

[43] Guertin DA, Sabatini DM (2007). Defining the role of mTOR in cancer. Cancer cell.

[44] Wang X, Du Z, Li L, Shi M, Yu Y (2015). Beclin 1 and p62 expression in non-small cell lung cancer: relation with malignant behaviors and clinical outcome. International journal of clinical and experimental pathology.

[45] Hug K, Grize L, Seidler A, Kaatsch P, Schuz J (2010). Parental occupational exposure to extremely low frequency magnetic fields and childhood cancer: a German case-control study. American journal of epidemiology.

[46] Tang R (2016). Extremely low frequency magnetic fields regulate differentiation of regulatory T cells: Potential role for ROS-mediated inhibition on AKT. Bioelectromagnetics.

[47] Ronchetto F (2004). Extremely low frequency-modulated static magnetic fields to treat cancer: A pilot study on patients with advanced neoplasm to assess safety and acute toxicity. Bioelectromagnetics.

[48] Liu YX (2012). Exposure to 1950-MHz TD-SCDMA electromagnetic fields affects the apoptosis of astrocytes via caspase-3-dependent pathway. PloS one.

[49] Gozuacik D, Kimchi A (2004). Autophagy as a cell death and tumor suppressor mechanism. Oncogene.

[50] Dalby KN, Tekedereli I, Lopez-Berestein G, Ozpolat B (2010). Targeting the prodeath and prosurvival functions of autophagy as novel therapeutic strategies in cancer. Autophagy.

[51] Kenific CM, Thorburn A, Debnath J (2010). Autophagy and metastasis: another double-edged sword. Current opinion in cell biology.

[52] Ye MX, Li Y, Yin H, Zhang J (2012). Curcumin: updated molecular mechanisms and intervention targets in human lung cancer. International journal of molecular sciences.

[53] Dang S (2015). Autophagy promotes apoptosis of mesenchymal stem cells under inflammatory microenvironment. Stem cell research & therapy.

[54] Zhang M, Su L, Xiao Z, Liu X, Liu X (2016). Methyl jasmonate induces apoptosis and pro-apoptotic autophagy via the ROS pathway in human non-small cell lung cancer. American journal of cancer research.

[55] Chatterjee A, Chattopadhyay D, Chakrabarti G (2014). miR-17-5p downregulation contributes to paclitaxel resistance of lung cancer cells through altering beclin1 expression. PloS one.

[56] Wei J (2015). miR-143 inhibits cell proliferation by targeting autophagy-related 2B in non-small cell lung cancer H1299 cells. Molecular medicine reports.

[57] Bhattacharya A (2015). miR-638 promotes melanoma metastasis and protects melanoma cells from apoptosis and autophagy. Oncotarget.

[58] Nian W (2013). miR-223 functions as a potent tumor suppressor of the Lewis lung carcinoma cell line by targeting insulin-like growth factor-1 receptor and cyclin-dependent kinase 2. Oncology letters.

[59] Huang L, Li F, Deng P, Hu C (2016). MicroRNA-223 Promotes Tumor Progression in Lung Cancer A549 Cells via Activation of the NF-kappaB Signaling Pathway. Oncology research.

[60] Liang H (2015). MicroRNA-223 delivered by platelet-derived microvesicles promotes lung cancer cell invasion via targeting tumor suppressor EPB41L3. Molecular cancer.

[61] Pang W (2014). Pim-1 kinase is a target of miR-486-5p and eukaryotic translation initiation factor 4E, and plays a critical role in lung cancer. Molecular cancer.

