# Supplementary material for: Low Frequency Magnetic Fields Induce Autophagy-associated Cell Death in Lung Cancer through miR-486-mediated Inhibition of Akt/mTOR Signaling Pathway
Source: Sci Rep. 2017 Sep 18;7:11776. doi: 10.1038/s41598-017-10407-w (PMC5603574; doi:10.1038/s41598-017-10407-w)
Supplement: Supplementary file 1 — supplementaryinformation [file 41598_2017_10407_MOESM1_ESM.pdf]

Supplementary Information

Low Frequency Magnetic Fields Induce Autophagy-associated Cell Death in Lung Cancer through miR-486-mediated Inhibition of Akt/mTOR Signaling Pathway

Yujun Xu [1], Yizhong Wang [1], Anran Yao [1], Zhen Xu [1], Huan Dou[1,2], Sunan Shen[1,2], Yayi Hou [1,2]*, Tingting Wang [1,2]*

1 The State Key Laboratory of Pharmaceutical Biotechnology, Division of Immunology, Medical School, Nanjing University, Nanjing 210093, China.
2 Jiangsu Key Laboratory of Molecular Medicine, Nanjing 210093, China.

* Correspondence to: Tingting Wang, The State Key Laboratory of Pharmaceutical Biotechnology, Division of Immunology, Medical School, Nanjing University, 22 Hankou Road, Nanjing 210093, China. Phone: 86-25-8368-6043; Fax: 86-25-8368-6043; E-mail: wangtt@nju.edu.cn; and Yayi Hou, The State Key Laboratory of Pharmaceutical Biotechnology, Division of Immunology, Medical School, Nanjing University, 22 Hankou Road, Nanjing 210093, China. Phone: 86-25-8368-6043; Fax: 86-25-8368-6043; E-mail: yayihou@nju.edu.cn

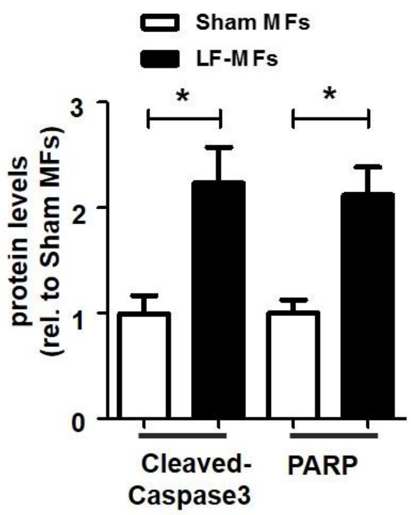

Figure S1. **LF-MFs suppress cell proliferation dependent of caspase 3 and PARP.**

Relative Cleaved-caspase 3 and PARP levels were determined by densitometry and normalized to each GAPDH protein levels. Experiments were repeated three times independently. Data represent mean ± S.D. (*p<0.05, **p<0.01).

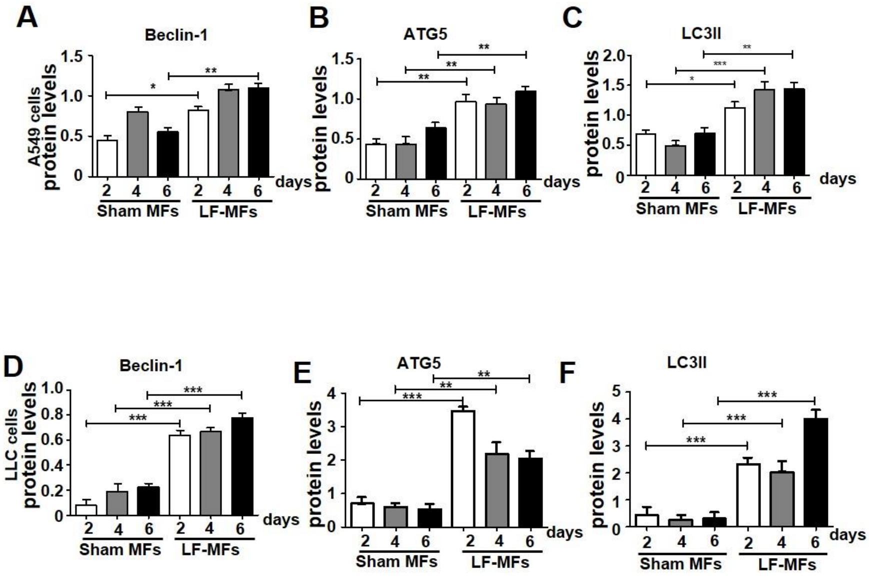

Figure S2. LF-MFs activates autophagy in lung cancer cells. (A-C) A549 cells and (D-F) LLC cells were treated with sham MFs or LF-MFs for different time intervals (2, 4, 6, days, 4h/day). Relative Beclin-1, ATG5 and LC3B levels were determined by densitometry and normalized to each GAPDH protein levels. Experiments were repeated three times independently. Data represent mean ± S.D. *p<0.05, **p<0.01, ***P < 0.001.

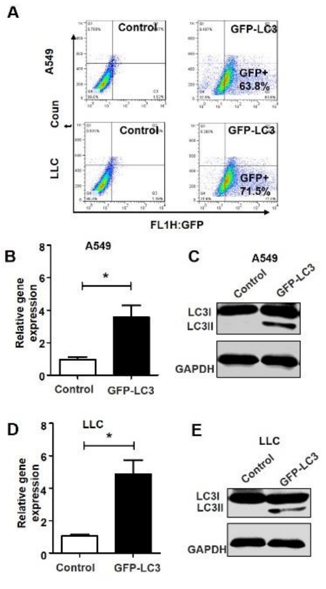

Figure S3. Generation of stable GFP-LC3 expressing A549 and LLC cell line. (A) A549 and LLC cells were transfected with GFP-control or GFP-LC3 plasmid. Flow cytometry analysis of the levels of GFP-LC3 compare with control in two cells. (B-E) Q-PCR and Western blot analysis the levels of LC3 showed in both A549 and LLC cells transfected with GFP-LC3 plasmid. Experiments were repeated three times independently. Data represent mean ± S.D. $*p<0.05$, $**p<0.01$.

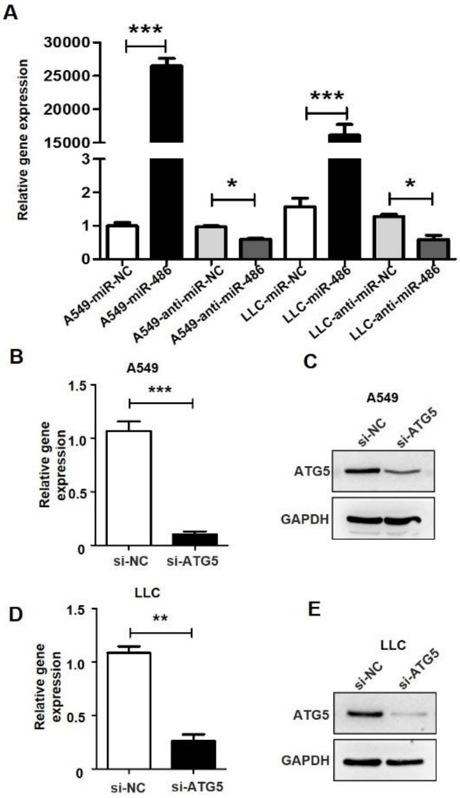

Figure S4. The effects of high-doses of miR-486 or miR-486 inhibition and the efficiency of si-RNA of ATG5 in two cell lines. (A) A549 and LLC cells were transfected 50 nM of miR-486 mimics, 100nM of miR-486 inhibitors or scrambled negative control for 48 h. The expression of miR-486 was analyzed by qPCR. (B-E) A549 and LLC cells were transfected with 50 nM of siNC or siATG5. The cells were harvested at 48 h after transfection. ATG5 mRNA level expression was determined by qPCR. ATG5 protein level was determined after transfection for 48 h by western blot and normalized to GAPDH. Experiments were repeated three times independently. Data represent means ± S. D. *p<0.05, **p<0.01, ****P*<0.001.

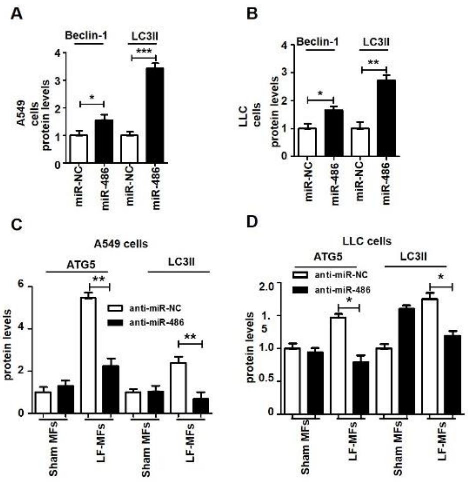

Figure S5. miR-486 is dependent for LF-MFs-induced cell autophagy. (A, B) A549 and LLC cells were transfected with 50 nM of scrambled negative control or miR-486 mimic. The cells were harvested at 48 h after transfection. Relative Beclin-1 and LC3II levels were determined by densitometry and normalized to each GAPDH protein levels. (C, D) A549 and LLC cells were transfected with miR-486 inhibiter or negative control. After 48 hours, cells were treated with sham MFs or LF-MFs. Relative ATG5 and LC3II levels were determined by densitometry and normalized to each GAPDH protein levels. Experiments were repeated three times independently. *P<0.05, ***P<0.001, ***P<0.001. Data represent means ± S. D.

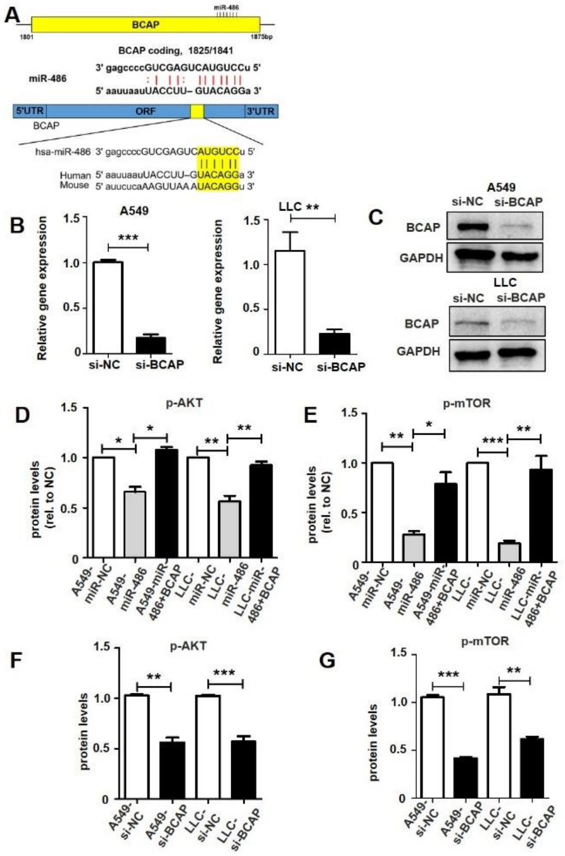

Figure S6. The miR-486 is highly binds to BCAP 3'UTR and inhibit Akt/mTOR pathway. (A) Schematic depicting the hypothetical duplexes formed by the interactions between the binding sites in the BCAP 3'-UTR and miR-486. (B, C) The dentification of siRNA for BCAP knockdown studies. A549 and LLC cells were transfected with 50 nM of siNC or siBCAP. The cells were harvested at 48 h after transfection. BCAP mRNA level expression was determined by qPCR. BCAP protein level determined by densitometry and normalized to each GAPDH protein levels. (D, E) BCAP plasmid was designed and was co-transfected with miR-NC or miR-486 mimic into A549 cells and LLC cells. Relative p-AKT and p-mTOR levels were determined by densitometry and normalized to each total protein levels. (F, G) A549 and LLC cells were transfected with 50 nM of siNC or siATG5. The cells were harvested at 48 h after transfection. Relative p-AKT and p-mTOR levels were determined by densitometry and normalized to each total protein levels. Experiments were repeated three times independently. **P*<0.05, ****P*<0.001, ****P*<0.001. Data represent means ± S. D.

Table S1. Primers used for real-time quantitative PCR analysis.

Gene	Sequence (5’to 3’)
has-LC3B-F	GATGTCCGACTTATTCGAGAGC
has-LC3B-R	TTGAGCTGTAAGCGCCTTCTA
has-Beclin1-F	CTGGTAGAAGATAAAACCCGGTG
has-Beclin1-R	AGGTAGAGCGTGGACTATCCG
has-ATG5-F	AAAGATGTGCTTCGAGATGTGT
has-ATG5-R	CACTTTGTCAGTTACCAACGTCA-3
has-BCAP-F	GAGCCAGAGACCTACGTGG
has-BCAP-R	TGTCATCCAGCTTACATCTCACA
has-GAPDH-F	GGAGCGAGATCCCTCCAAAAT
has-GAPDH-R	GGCTGTTGTCATACTTCTCATGG
has-miR-486-5p-F	ACACTCCAGCTGGG TCCTGTACTGAGCTGC
has-miR-486-5p-R	CTCAACTGGTGTCGTGGAGTCGGCAATTCAGTTGAGCT CGGGGC
mmu-GAPDH-F	ATCTCCGCCCCTTCTGCCGA
mmu-GAPDH-R	CCACAGCCTTGGCAGCACCA
mmu-LC3B-F	TTATAGAGCGATACAAGGGGGAG
mmu-LC3B-R	CGCCGTCTGATTATCTTGATGAG
mmu-ATG5-F	TGTGCTTCGAGATGTGTGGTT
mmu-ATG5-R	ACCAACGTCAAATAGCTGACTC
mmu-BCAP-F	GTCCCGGATGCCTCTTTCTC
mmu-BCAP-R	CACAAGTCATTTCCTGCCAGT
mmu-miR-486-5p-F	ACACTCCAGCTGGG TCCTGTACTGAGCTGC
mmu-miR-486-5p-R	CTCAACTGGTGTCGTGGAGTCGGCAATTCAGTTGAGCT CGGGGC

Western Blot original date

Figure.1

H

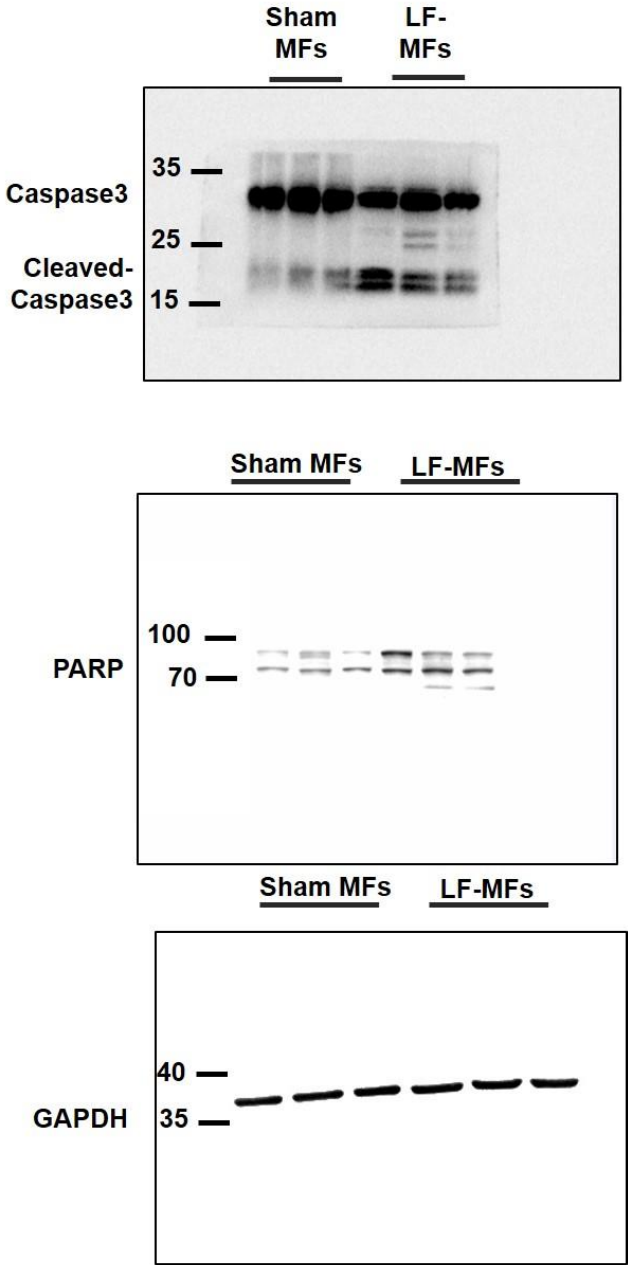

Figure. 2

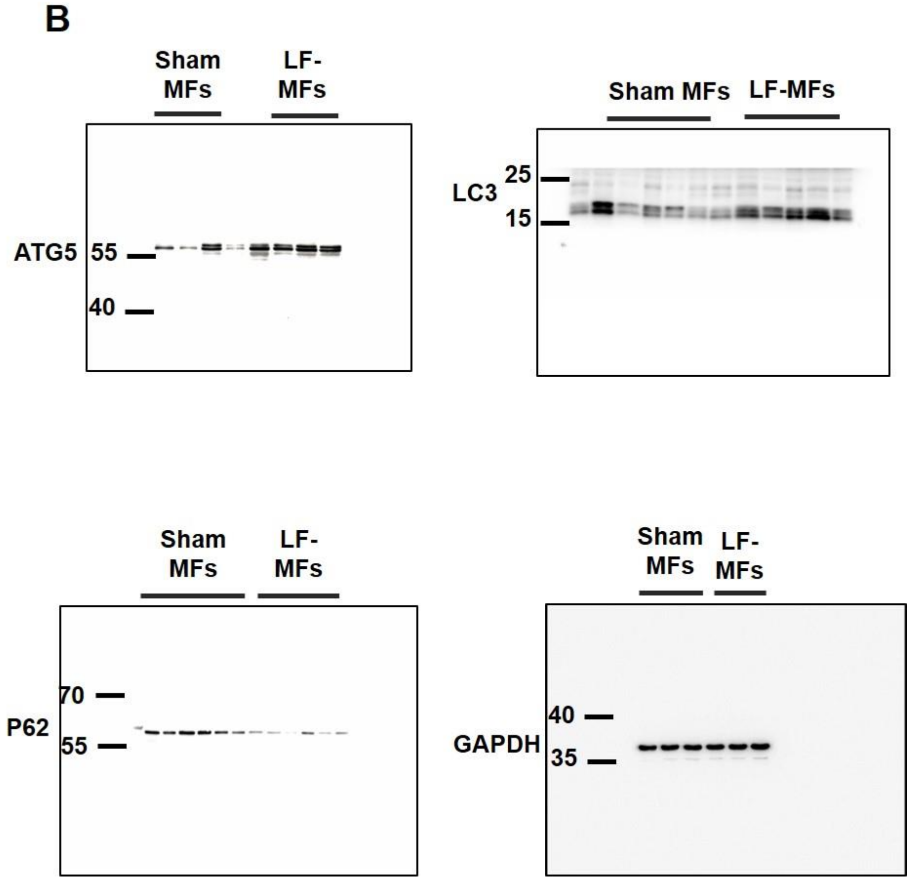
B
Sham MFs
LF-MFs
ATG5
55
40
Sham MFs
LF-MFs
LC3
25
15
Sham MFs
LF-MFs
P62
70
55
Sham MFs
LF-MFs
GAPDH
40
35

Figure. 2

D

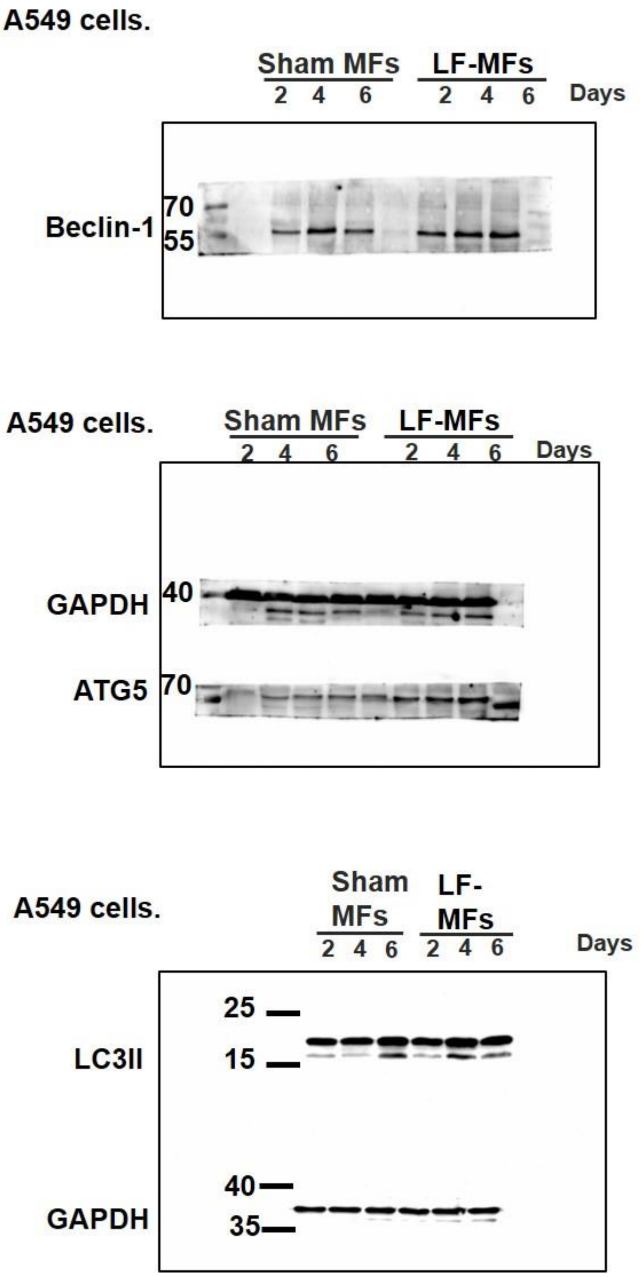
A549 cells.
Sham MFs
LF-MFs
2 4 6
2 4 6
Days
Beclin-1
70
55
A549 cells.
Sham MFs
LF-MFs
2 4 6
2 4 6
Days
GAPDH
40
ATG5
70
A549 cells.
Sham MFs
LF-MFs
2 4 6
2 4 6
Days
25
LC3II
15
40
GAPDH
35

Figure. 2

E

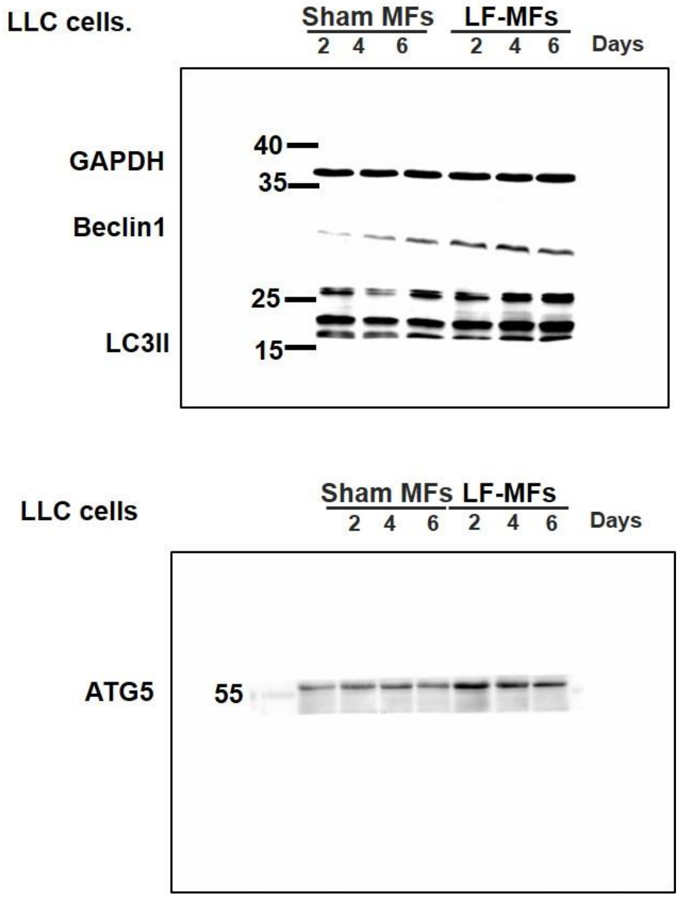
LLC cells.
Sham MFs
LF-MFs
2 4 6 2 4 6 Days
GAPDH
Beclin1
LC3II
40
35
25
15
LLC cells
Sham MFs LF-MFs
2 4 6 2 4 6 Days
ATG5
55

Figure. 5

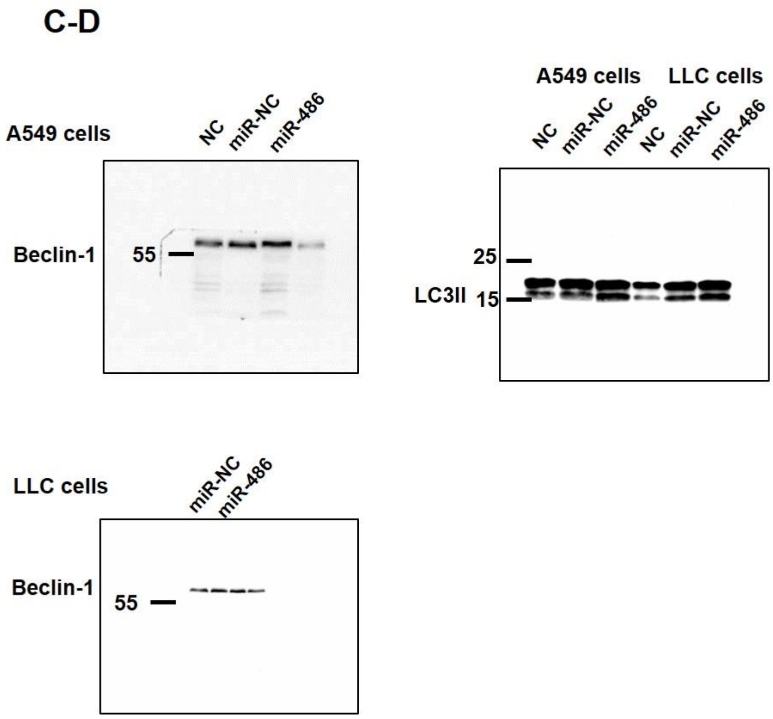
C-D
A549 cells
NC
miR-NC
miR-486
Beclin-1
55
A549 cells
LLC cells
NC
miR-NC
miR-486
NC
miR-NC
miR-486
25
LC3II
15
LLC cells
miR-NC
miR-486
Beclin-1
55

Figure. 5

G

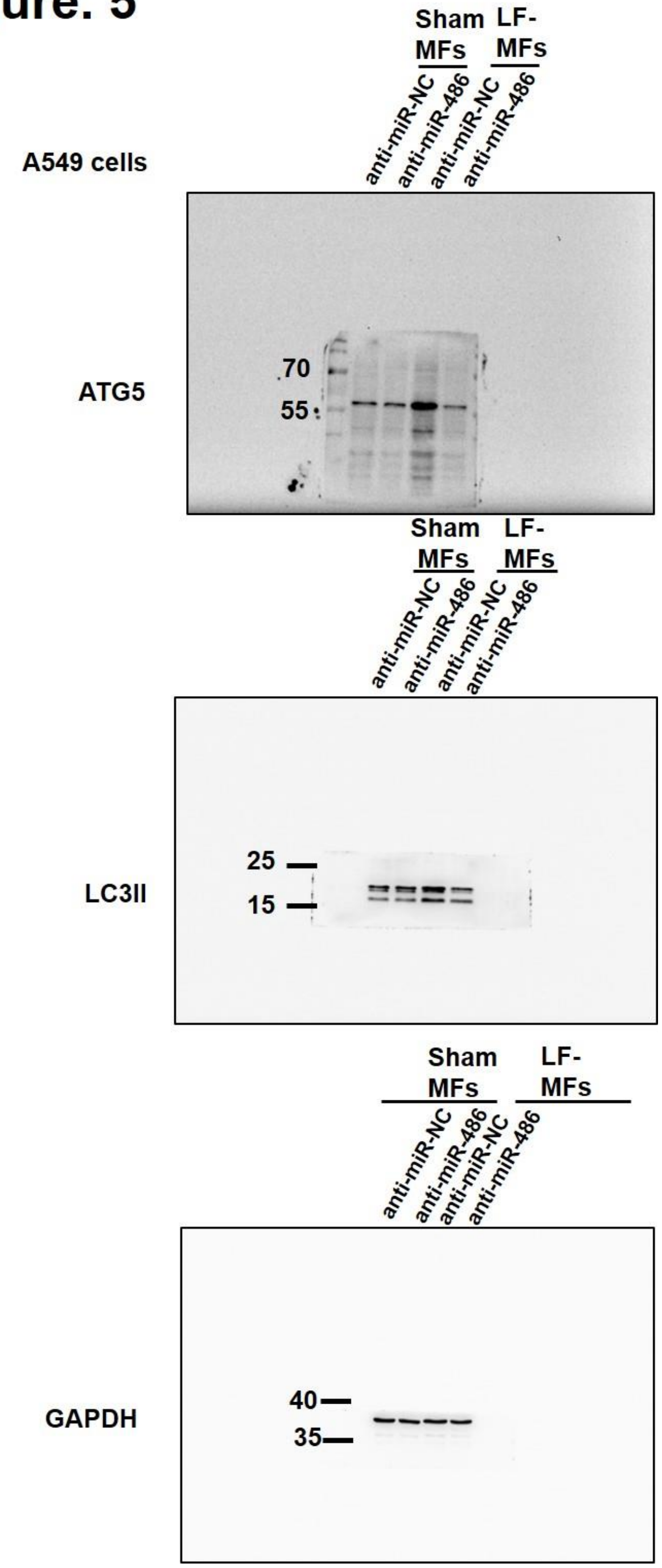
A549 cells
Sham MFs
LF-MFs
anti-miR-NC
anti-miR-486
anti-miR-NC
anti-miR-486
ATG5
70
55
Sham MFs
LF-MFs
anti-miR-NC
anti-miR-486
anti-miR-NC
anti-miR-486
LC3II
25
15
Sham MFs
LF-MFs
anti-miR-NC
anti-miR-486
anti-miR-NC
anti-miR-486
GAPDH
40
35

Figure. 5

H LLCcells

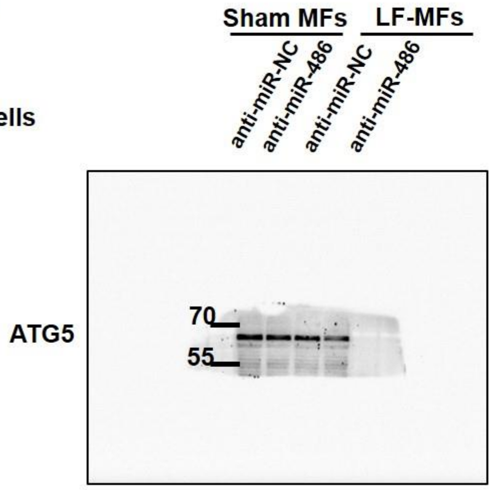

LLCcells

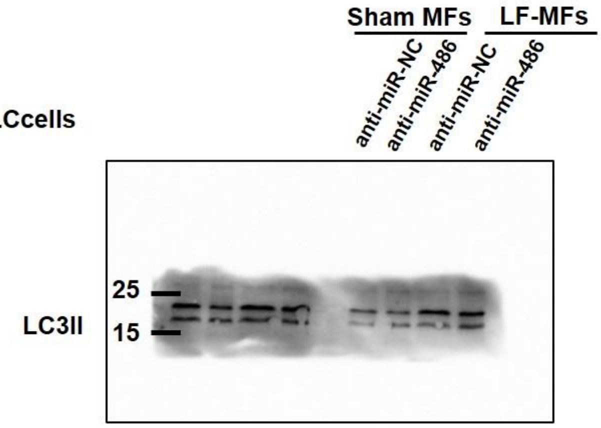

LLCcells

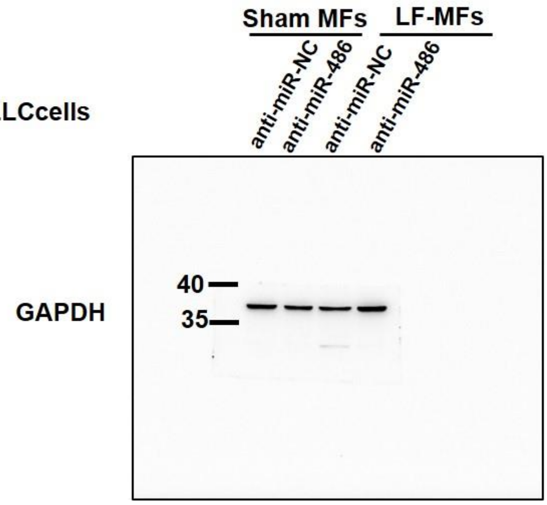

Figure. 6

E

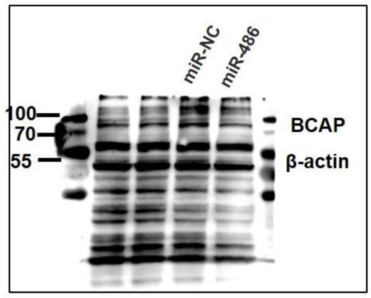
miR-NC
miR-486
100
70
55
BCAP
β-actin

F

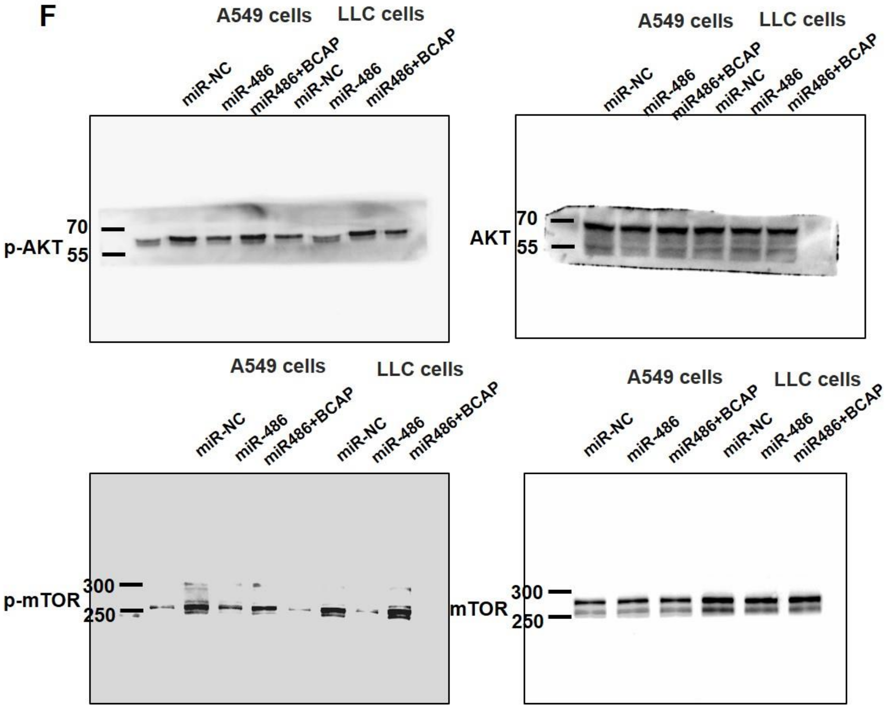
A549 cells
LLC cells
miR-NC
miR-486
miR486+BCAP
miR-NC
miR-486
miR486+BCAP
p-AKT
70
55
A549 cells
LLC cells
miR-NC
miR-486
miR486+BCAP
miR-NC
miR-486
miR486+BCAP
AKT
70
55
A549 cells
LLC cells
miR-NC
miR-486
miR486+BCAP
miR-NC
miR-486
miR486+BCAP
p-mTOR
300
250
A549 cells
LLC cells
miR-NC
miR-486
miR486+BCAP
miR-NC
miR-486
miR486+BCAP
mTOR
300
250

Figure. 6

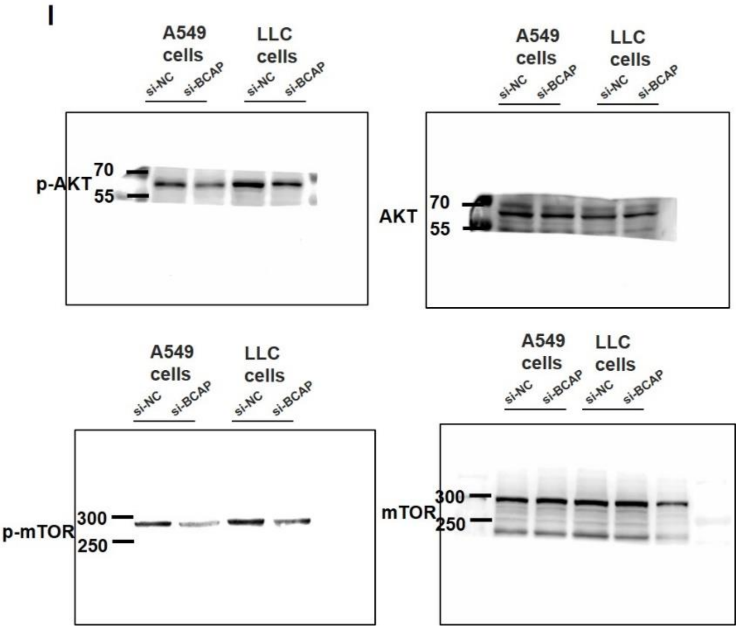
I
A549 cells
LLC cells
si-NC
si-BCAP
si-NC
si-BCAP
p-AKT
70
55
A549 cells
LLC cells
si-NC
si-BCAP
si-NC
si-BCAP
AKT
70
55
A549 cells
LLC cells
si-NC
si-BCAP
si-NC
si-BCAP
p-mTOR
300
250
A549 cells
LLC cells
si-NC
si-BCAP
si-NC
si-BCAP
mTOR
300
250

Figure. S3

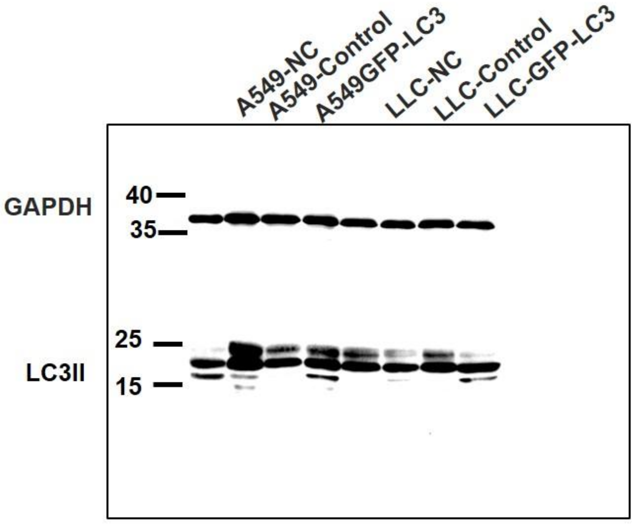

Figure. S5

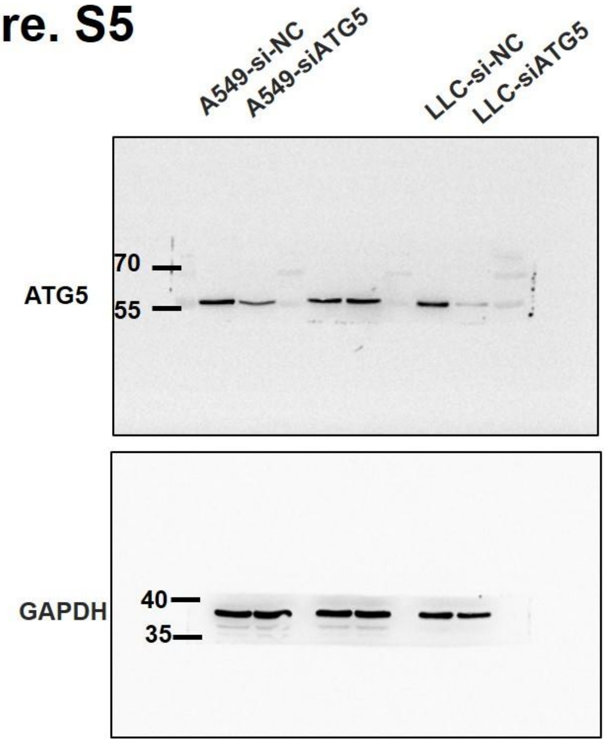

Figure. S7

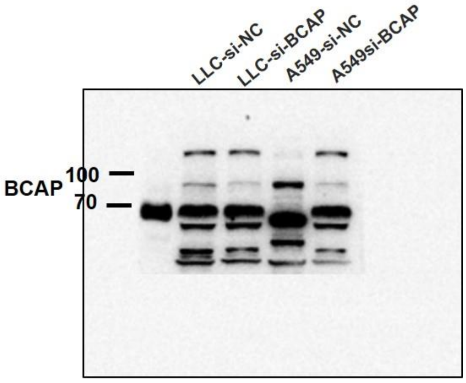
LLC-si-NC
LLC-si-BCAP
A549-si-NC
A549si-BCAP
BCAP
100
70

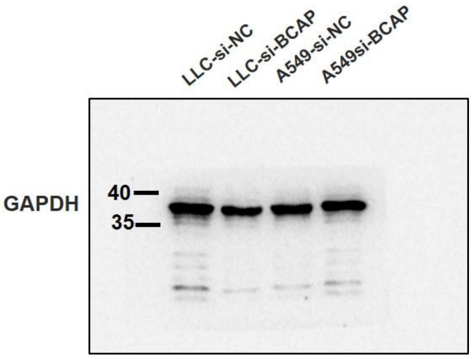
LLC-si-NC
LLC-si-BCAP
A549-si-NC
A549si-BCAP
GAPDH
40
35
